# Structure-function relationships in the nasal cavity of Arctic and subtropical seals

**DOI:** 10.1016/j.bpj.2023.11.012

**Published:** 2023-12-14

**Authors:** Hyejeong L. Cheon, Signe Kjelstrup, Nataliya Kizilova, Eirik G. Flekkøy, Matthew J. Mason, Lars P. Folkow

**Affiliations:** 1 PoreLab, Department of Physics, Norwegian University of Science and Technology, NTNU, Trondheim, Norway; 2 PoreLab, Department of Chemistry, Norwegian University of Science and Technology, NTNU, Trondheim, Norway; 3 V.N. Karazin Kharkov National University, Kharkov, Ukraine; 4 PoreLab, Department of Physics, University of Oslo, Oslo, Norway; 5 Department of Physiology, Development & Neuroscience, University of Cambridge, Cambridge, UK; 6 Department of Arctic and Marine Biology, UiT – The Arctic University of Norway, Tromsø, Norway

## Abstract

The heating and moistening of inhaled air, and the cooling and moisture removal from exhaled air, are crucial for the survival of animals under severe environmental conditions. Arctic mammals have evolved specific adaptive mechanisms to retain warmth and water and restrict heat loss during breathing. Here, the role of the porous turbinates of the nasal cavities of Arctic and subtropical seals is studied with this in mind. Mass and energy balance equations are used to compute the time-dependent temperature and water vapor profiles along the nasal passage. A quasi-1D model based on computed tomography images of seal nasal cavities is used in numerical simulations. Measured cross-sectional areas of the air channel and the perimeters of the computed tomography slices along the nasal cavities of the two seal species are used. The model includes coupled heat and vapor transfer at the air-mucus interface and heat transfer at the interfaces between the tissues and blood vessels. The model, which assumes constant blood flow to the nose, can be used to predict the temperature of the exhaled air as a function of ambient temperature. The energy dissipation (entropy production) in the nasal passages was used to measure the relative importance of structural parameters for heat and water recovery. We found that an increase in perimeter led to significant decreases in the total energy dissipation. This is explained by improved conditions for heat and water transfer with a larger complexity of turbinates. Owing to differences in their nasal cavity morphology, the Arctic seal is expected to be advantaged in these respects relative to the subtropical seal.

## Significance

We show here that the Arctic seal *Erignathus barbatus* has a lower respiratory energy loss than the subtropical seal *Monachus monachus,* when both are exposed to the same conditions. We show, using a dynamic model for the breathing cycle in the maxilloturbinate region, that the nasal cavity structure, expressed by the cross-sectional area of the air channel and the perimeter of this area, determines the energy efficiency of the heat and mass transfer. The structure of the Arctic seal nasal cavity, in particular the maxilloturbinate perimeter, helps the animal to reduce the energy dissipation 23% below that of the subtropical seal. Owing to differences in their nasal cavity morphology, the Arctic seal is expected to be advantaged in colder climates relative to the subtropical seal.

## Introduction

The structure-function relationships of tissues and organs of mammals are topics of wide interest (1,2,3,4). Arctic mammals can survive under extreme environmental conditions (low temperatures, limited food and water), possibly because they have evolved various anatomical structures and specialized physiology that allow them to reduce and control their energy dissipation, thereby maintaining their body heat and water losses at a low level (2,3,5). Heat is lost in a cold climate, not only through the skin, but also during breathing itself, by heat and moisture transfer from the animal to the surroundings.

The mammalian nasal cavity contains the maxilloturbinate (MT) bones; one on each side. The MTs have a complex structure known to play an important role in body heat exchange (1,6,7,8,9,10). When the cold air passes through the nasal cavity upon inhalation, it warms gradually until it reaches the core body temperature as found in the lung. In this process, the air also becomes saturated with water vapor. This conditioning of the air is important for proper lung function (1,11,12). Upon exhalation, it is unavoidable that some of this heat and water is lost, but adsorption will also take place from the air back into the tissues, reducing the heat and water losses to the environment. This reduction depends on ambient conditions as well as the particular structure of the MT bones (6,7,8,9,10,11,13,14,15).

Mathematical models of heat and mass transfer in the repetitive cycles of inhalation-exhalation may allow a better understanding of the physical processes involved in this exchange. A simplified biophysical model of the steady-state respiratory heat transfer was thus developed in the kangaroo rat, using the mass and energy balances for airflow between parallel plates and a concurrent flow of blood at a constant body temperature ($T_{\text{body}}$) (16). Humidification of the inhaled air was described by the diffusion equation for water vapor. Anatomical and physiological features related to the efficiency of nasal heat exchange have been studied in several mammals, including camels (17), goats (18), koala (19), dogs (20), humans (11,12), and reindeer (9,13).

In a recent study (14), it was found that both Arctic and Antarctic seals have a nasal cavity with larger total cross-sectional area, containing more complex MTs than subtropical seals. This supports the idea that the nasal structures in polar seals have evolved in response to cold environmental conditions. Flekkøy et al. (21) modeled the countercurrent heat exchange along the nose, assuming the air to always be saturated with vapor. In this model (called model I in this paper) there was also a rapid exchange of heat with the blood that perfuses the MT mucosa, meaning that there was a significant amount of heat transfer in both directions to and from the blood. The blood flow was adjusted to give the experimental nose temperature. Model I did not investigate the dynamics of the heat and mass exchange and therefore the effect of a rate-limiting water condensation or evaporation at air-tissue interfaces. Also, model I did not include varying geometrical properties of the air passage within the nasal cavity. In the more sophisticated model presented here, model II, we therefore study water and heat exchange of the full breathing cycle with a varying cross-sectional airflow area and perimeter along the nose, and without any adjustment of blood flow, but at set airflow inlet and outlet temperatures. In model II we do *not* assume that the air is fully saturated with water vapor.

Our work concerns a part of the respiratory tract, the MT section, which is responsible for proper heating/cooling and moistening/demoistening of the inhaled/exhaled air at varying ambient temperature and humidity. We study the effect of varying geometry of the MT under various nonequilibrium conditions and the performance of this organ in different species. The remaining part of the respiratory tract will thus be kept constant; i.e., the processes in the pharynx, trachea, and bronchial part of the seals’ respiratory tract are assumed to be the same when we compare the species. The seal turbinate structure is so much more complicated than that of humans that it seems likely that the MT system is able to condition the air much more fully before it reaches the bronchial tree (22). This allows us to restrict the attention to the MT system alone.

We use model II to find the total entropy production, $\Sigma_{\text{irr}}$, of a quasi-3D model of the nasal cavity (model II) to measure variations in the energy efficiency of the nasal cavity function. This property has already been used as an efficiency measure of the respiratory systems of mammals and humans (6,23,24). The entropy production due to viscous dissipation in human lungs was calculated at various levels of humidity and branching of the bronchial tree of the lungs (23,24,25). The entropy production per day, measured for the resting state, was 50 kJ/K (24). Values depended on the ambient temperature and the relative humidity of the ambient air (23). In the human respiratory system, an analysis of the dissipated energy has shown an efficiency decrease of 21 or 16.5% for moderate or extreme levels of activity, respectively, compared with that of the resting state (26). Clearly, the energy budget of animals is a vital physiological consideration, but existing studies suggest that there will be differences in the efficiency of nasal heat exchange according to species and circumstances. We therefore use model II to compute the entropy production of the breathing cycle, to compare structure-function relationships in Arctic and subtropical seals. The aim of the work is to understand better the role of MT complexity in the functions of the nasal cavity under extreme environmental conditions.

The paper is structured as follows. In the methods and materials, we recapitulate the anatomical and functional properties needed to model relevant seal noses. In model II, we present the details of the nonequilibrium thermodynamic model in use, as applied to the seal nose. We document the numerical solution procedure, give an overview of the cases studied, and compare the model with experimental results as far as possible. The numerical results are interpreted and analyzed in results and discussion. A video showing the breathing dynamics is included in the supporting material. We hypothesize that the complexity of the nasal cavity in an Arctic seal, here represented by *Erignathus barbatus* (Eb), enables this animal to function with lower energy dissipation (entropy production) than the subtropical seal, *Monachus monachus* (Mm), when both are placed under Arctic conditions. Concluding remarks follow.

## Methods and materials

### Structure of the MT region

**Figure 1:**
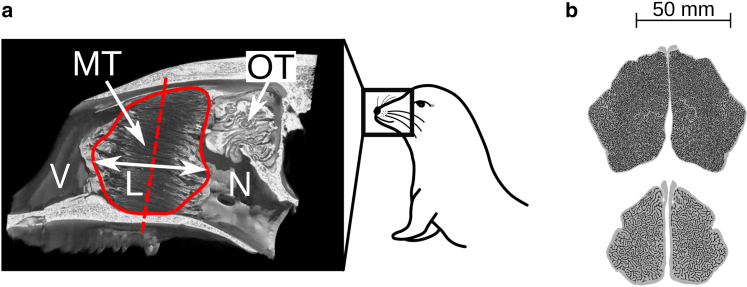
Structure of the nasal cavities of the Arctic seal (Eb) and the subtropical seal (Mm). (*a*) The longitudinally sectioned CT reconstruction of the Arctic seal’s skull with maxilloturbinates (MTs) of length *L* in the anteroposterior direction (contained within the solid red line) and olfactory turbinates (OTs). The inhaled air passes from the nostrils (not visible) through an open, vestibular region (V), through the MTs, and then through the nasopharynx (N). (*b*) Cross-sectional CT images of MT of the Arctic seal (*top*) and the subtropical seal (*bottom*) in approximately the position marked by the red dashed line in (*a*). Scale bar, 50 mm. CT images are reproduced with permission of Mason et al. (14), Copyright 2020 Elsevier. To see this figure in color, go online.

The geometry of the bony part of the nasal cavity is known from computed tomography (CT) images of the seal’s skull (see Fig. 1 *a*). The nasal cavity starts at the vestibule, which lies just behind the nostrils. From here, the air passes through the MT and the nasopharynx, ultimately reaching the lungs. Fig. 1 *b* shows CT cross sections of the most complex region of MT in the bearded seal (Eb) and the Mediterranean monk seal (Mm). We call the Arctic seal Eb and the subtropical seal Mm for convenience.

**Figure 2:**
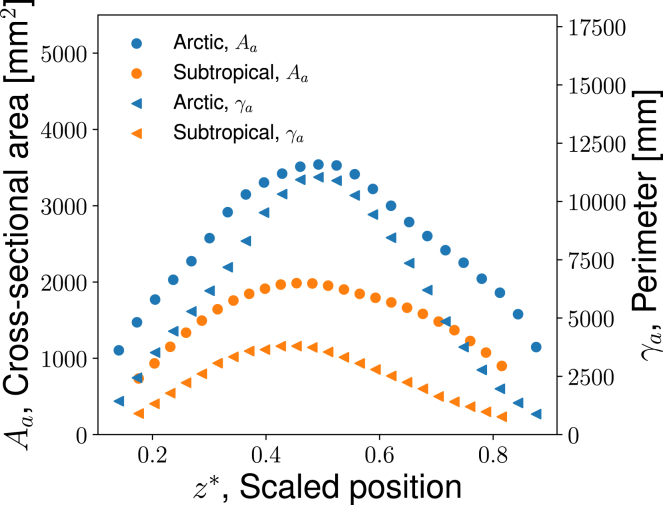
The cross-sectional area ($A_{a}$) of the channel for airflow and the corresponding perimeter ($\gamma_{a}$) for the Arctic (Eb) and the subtropical (Mm) seals, as computed from CT data. Both variables are plotted as a function of scaled position, $z^{*}=z/L$, where *L* is the length of the MT region. Here, $z^{*}=0$ and $z^{*}=1$ refer to the distal (the nostrils) and proximal ends of the MT region, respectively. To see this figure in color, go online.

The 3D volume of the MT was divided into 164 slices for the Arctic seal (Eb) and 172 slices for the subtropical seal (Mm) along the *z* axis in the anteroposterior direction, and a corresponding number of 2D slices (like those presented in Fig. 1 *b*) were obtained. For each slice, we measured the cross-sectional area available for airflow, $A_{a}$. This is the area contained within the MT mass that is shown by gray pixels in Fig. 1 *b*. The corresponding perimeter of the air-tissue interface, $\gamma_{a}$, is also shown. The value of $\gamma_{a}$ multiplied by the thickness of the slice in the length direction gives the wall surface area for heat and water exchange at the location in question. The scans were of dried seal skulls; all measurements refer to dry MT bone (14), so $A_{a}$ is technically speaking the cross-sectional area of the non-bony components in the MT. The measured results for $A_{a}$ and $\gamma_{a}$ of each section are shown in Fig. 2 as a function of scaled position $z^{*}=z/L$. $A_{a}\left(z^{*}\right)$ and $\gamma_{a}\left(z^{*}\right)$ vary along the nose, with maximum values located approximately in the center of the MT. *L* is the length of the MT. A nondimensional parameter, $z^{*}$, was used to indicate position, because *L* differs between both individuals and seal species.

$A_{a}$ and $\gamma_{a}$ had their minimum values near the ends, near $z^{*}=0$ and $z^{*}=1$ (see Fig. 2, *a* and *b*). Beyond these values, we assumed that no significant heat and water exchange took place. $A_{a}$ reduces to zero at the start and the end because the MTs taper off there. However, the total area for airflow remains high at both ends, because there is space between the wall and the MT mass here. This is visible in Fig. 1 *a*. The airflow pathway outside the MT plays no role in heat and water exchange in the model, however, and is therefore ignored in this context.

In reality, the MT bone is not dry, but covered by soft tissue (mucosa), including epithelial cells, interstitial tissues with blood vessels, plus a liquid-containing mucus layer (27). This results in some inaccuracy in $A_{a}\left(z^{*}\right)$ and $\gamma_{a}\left(z^{*}\right)$. The trends in the curves of Fig. 2 should capture the true situation well, however. The data selected for analysis are represented by points in the range $z^{*}=0.18\;–\;0.82$ for the subtropical seal (Mm) and $z^{*}=0.14\;–\;0.87$ for the Arctic seal (Eb) (see Fig. 2). In the figures where we present results, these points define the endpoints and the length of the MT.

The measured variations in $A_{a}\left(z^{*}\right)$ and $\gamma_{a}\left(z^{*}\right)$ were used to create a quasi-3D model of the nasal passages, as was done earlier in reindeer (6). Instead of an airway with complex cross-sectional geometry (Fig. 2), we created a model tube that has the (varying) cross-sectional area for airflow $A_{a}\left(z\right)$ and perimeter $\gamma_{a}\left(z\right)$ along the *z* axis. This tube will be used for numerical computations for different boundary conditions. A continuous blood flow supplies all tissues in the nasal cavity, thus providing heating and moistening of the inhaled cold air. The contribution from the blood here is implicitly accounted for by the chosen boundary conditions.

### Model II

We constructed model II to study the system away from equilibrium since model I (21) assumes equilibrium at each position in the MT. The governing equations of our model II for the seal nose are area-averaged balance equations for mass and energy. Readers are referred to Jakobsen for further details (28, p. 12–60). The equations for coupled transport of heat and water at the mucus lining were further formulated according to nonequilibrium thermodynamics (29). Details and derivations of these equations are available (6). The equations are repeated for convenience in the supporting material, section 1 (Eqs. S1–S42).

**Figure 3:**
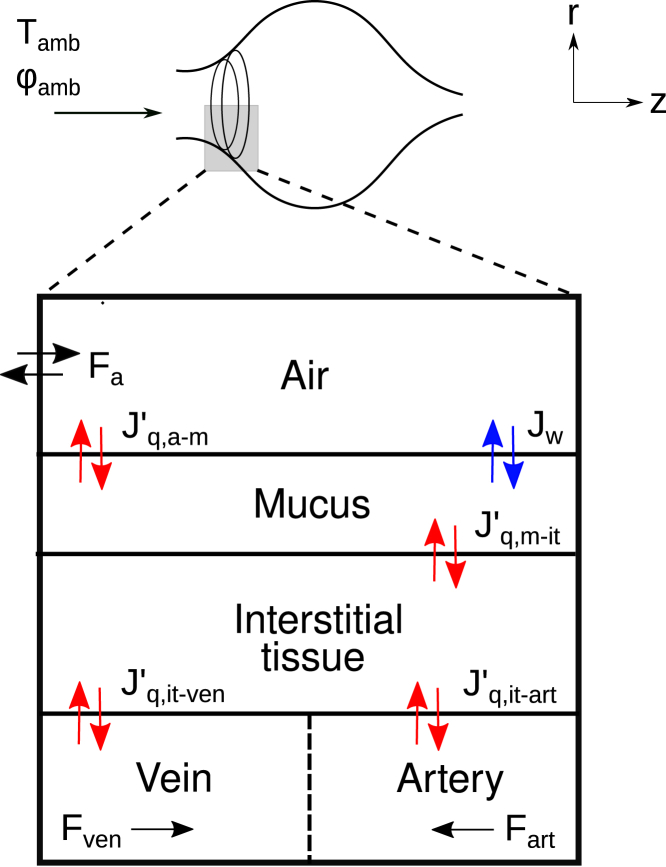
Subsystems of the model: air enters with ambient temperature $T_{\text{amb}}$ and relative humidity $\varphi_{\text{amb}}$. The nasal cavity with air is denoted (a), the liquid mucus layer (m), the interstitial tissue (it), vein (ven), and artery (art). Red and blue arrows indicate the heat and water fluxes, respectively, while black arrows indicate the flow of air and blood. All fluxes are in the *r*-direction and flows are in the *z*-direction. For a definition of fluxes, see text. To see this figure in color, go online.

Model II has a compartmental system as described in Fig. 3, in agreement with earlier work (6,7). The nasal cavity is modeled by five subsystems as pictured in this figure: the air passage (a), the liquid mucus (m), the interstitial tissue (it), the arteries (art), and the veins (ven). Fig. 3 shows the heat measurable fluxes, $J_{q,a-m}^{\prime }$, $J_{q,m-\text{it}}^{\prime }$, $J_{q,\text{it}-\text{art}}^{\prime }$, $J_{q,\text{it}-\text{ven}}^{\prime }$, and the mass flux, $J_{w}$. These fluxes are all in the radial direction. The air flow, artery flow, and the vein flow, $F_{a}$, $F_{\text{art}}$, and $F_{\text{ven}}$, are all in the axial direction (27). The direction from subsystem *i* to subsystem *j* is indicated by $J_{i-j}$. For example, $J_{q,a-m}^{\prime }$ is the measurable heat flux from the air (a) to the mucus (m). The flux $J_{w}$ is positive in the direction from the air to the mucus. In other words, if $J_{w}$ is positive, this means that there is condensation on the mucus lining and, if it is negative, evaporation takes place from the mucus lining. The flux $J_{m}$ is the mass flux of liquid water from the underlying tissue to the mucus lining. This is not shown in Fig. 3. Heat and water transfer between the various nasal compartments were modeled using the following assumptions:

1) The composition of unsaturated air (with 90% relative humidity) is constant during the breathing cycle. The density of the air, $\rho_{a}$, is determined by the temperature, pressure, and water mole fraction and the Peng-Robinson equation of state (30).
2) The density of the mucus layer is constant and equal to the density of water. The density of the interstitial tissue is constant and is the same as the density of blood. The density of blood in arteries and veins, blood pressure, and blood flow rate are constant.
3) The metabolic heat production from all cells in the tissue of the system is ignored.
4) The pressure variation along the airflow channel is neglected. Flekkøy et al. (21) computed the pressure drop, which should be overcome by the respiratory muscles and the corresponding power (work per unit time). This is less than 0.7 W for both seals.
5) The volume fractions of arteries, veins, and interstitial tissue in the seal’s nose are modeled in the same way as for reindeer (6,13). Details are given in the supporting material, section 1.
6) Airflows through the right and left MT masses took place over the length modeled. No flow took place between the MT and the nasal cavity walls.

All variables are computed for a cross section assuming radial symmetry. A variable is thus given as an average over the cross-sectional area along the length of the cylinder in the axial direction of the nasal cavity. This is a reasonable way to describe 3D variables by a 1D model (31,32)—which is then a quasi-1D model. The complexity of the nasal turbinates was expressed by the dimensionless number, $\delta^{*}=\gamma_{a}/\sqrt{A_{a}}$, where $\gamma_{a}$ is the perimeter and $A_{a}$ is the cross-sectional area of the airspace (7).

The breathing of the seal in the resting state was assumed to be periodical (6,33). A sinusoidal function was therefore used to model the breathing.

(1) $$F_{a}=F_{a,\max}\;\sin\;2\;\pi \left(\frac{t}{t_{\text{br}}}\right)$$

Here, $F_{a,\max}=\left(\rho_{a}V_{t}/t_{\text{br}}\right)$ where *t* is the time, $t_{\text{br}}$ is the duration of a single breathing cycle, $V_{t}$ is the tidal volume, and $\rho_{a}$ is the density of the air. For convenience, we use a dimensionless time variable $t^{*}=t/t_{\text{br}}$. The values $t_{\text{br}}$ and $V_{t}$ are given in Table 1. Data for $t_{\text{br}}$ are not available for the resting state, so we used data for active, swimming seals (34). While the estimated amplitude of the breathing signal may be reasonable, the frequency may be too high, leading to an overestimation of the energy dissipation.

**Table 1:** Geometrical and other data of the Arctic and subtropical seals

Symbol	Description	Arctic seal	Subtropical seal	Unit	Reference
		(Eb)	(Mm)		
*L*	MT length	6.10 × 10^–2^	5.16 × 10^–2^	m	–
$A_{a,\max}$	max. cross-sectional area	3.55 × 10^–3^	1.99 × 10^–3^	m^2^	–
$\gamma_{a,\max}$	max. perimeter	11.1	3.82	m	–
$d_{m}$	mucus thickness	1 × 10^–5^	1 × 10^–5^	m	(40)
$T_{\text{body}}$	core body temperature	36	36	°C	(15,22)
$T_{\text{amb}}$	ambient temperature	−30.0	5.0/10.0/15.0	°C	(15,22)
$\varphi_{\text{body}}$	relative humidity of deep body	100	100	%	(6)
$\varphi_{\text{amb}}$	relative humidity of ambient air	90	90	%	(6)
*M*	body mass	180	180	kg	(41,42)
$V_{t}$	tidal volume	6.3	6.3	L	(34)
$t_{\text{br}}$	duration of a single breathing cycle	3.09	3.09	s	(34)

The $A_{a}$ and $\gamma_{a}$ vary with scaled position, $z^{*}$, so we present maximum values in this table, $A_{a,\max}$ and $\gamma_{a,\max}$. For further definitions of symbols, see text.

Boundary conditions were taken to be similar to those used for reindeer in a previous study (6). The water mass fraction $w_{a}$ was calculated from the ambient temperature $T_{\text{amb}}$. The water mass fraction is the ratio of water vapor mass to the total mass in the air, and this is used as a direct indicator to show how much water vapor is contained in the expired air from the lungs. The relative humidity RH $=\varphi_{\text{amb}}$ was assumed constant at the distal end ($z^{*}=0$) during inhalation, and at the proximal end ($z^{*}=1$) during exhalation. In the reference set of data, the relative humidity of the ambient air is 90%, $\varphi_{\text{amb}}=90$%, at the inlet (see Table 1), the saturation value at $z^{*}=1$, $\varphi_{\text{body}}=100\%$. The data were taken for lung conditions given in Table 1. During inhalation and exhalation, heat is also supplied from or returned to the arteries, so as to maintain the temperature gradient in the channel. The temperature of arteries at $z^{*}=1$ was set as $T_{\text{body}}$. Blood in the arteries and veins exchange heat through capillaries in the interstitial tissue. On this basis, we give $T_{\text{art}}$ at $z^{*}=0$ as $T_{\text{ven}}$ at $z^{*}=0$. The temperature of the mucus layer and interstitial tissue at $z^{*}=1$ is also set as $T_{\text{body}}$. This can be summarized as follows:

During inhalation,

$$\varphi_{a}\left(z^{*}=0\right)=\varphi_{\text{amb}}\text{.}$$

$$T_{a}\left(z^{*}=0\right)=T_{\text{amb}}\text{.}$$

$$T_{m}\left(z^{*}=1\right)=T_{\text{body}}\text{.}$$

$$T_{\text{it}}\left(z^{*}=1\right)=T_{\text{body}}\text{.}$$

$$T_{\text{art}}\left(z^{*}=1\right)=T_{\text{body}}\text{.}$$

$$T_{\text{ven}}\left(z^{*}=0\right)=T_{\text{art}}\left(z^{*}=0\right)\text{.}$$

During exhalation,

$$\varphi_{a}\left(z^{*}=1\right)=\varphi_{\text{body}}\text{.}$$

$$T_{a}\left(z^{*}=1\right)=T_{\text{body}}\text{.}$$

$$T_{m}\left(z^{*}=1\right)=T_{\text{body}}\text{.}$$

$$T_{\text{it}}\left(z^{*}=1\right)=T_{\text{body}}\text{.}$$

$$T_{\text{art}}\left(z^{*}=1\right)=T_{\text{body}}\text{.}$$

$$T_{\text{ven}}\left(z^{*}=0\right)=T_{\text{art}}\left(z^{*}=0\right)\text{.}$$

### Energy dissipation (entropy production)

The total entropy production of a process can be regarded as originating from the (generalized) friction of the process. Whenever there are gradients in temperature or composition, there is friction associated with the attempt to reduce the gradients. Some of the total energy is dissipated in this process. This energy is lost as heat, it can no longer be used for work, and is therefore also called lost work. Evaporation at saturated conditions is an equilibrium process with zero friction. The dissipation can be observed by heating in the surroundings. The dissipated energy is the entropy production times the temperature of the surroundings, $W_{\text{lost}}=T_{a}\Sigma_{\text{irr}}$. Such losses must be compensated by the energy obtained from food.

The dissipation must not be confused with heat flow from one part to another. Heat flow can occur without losses when a process like condensation occurs at equilibrium conditions. The full phrase is *energy dissipated as heat to the surroundings*. The contributions to the total entropy production from each subsystem are $\Sigma_{a-m}$, $\Sigma_{m-\text{it}}$, $\Sigma_{\text{it}-\text{art}}$, and $\Sigma_{\text{it}-\text{ven}}$. The entropy production of an area element for heat and mass exchange in Fig. 3, is defined by the product sum of conjugate thermodynamic driving forces, $X_{i}$, and fluxes, $J_{i}$ (35). For the cross-sectional area, the local entropy production is obtained by integration of the area:

(2) $$\sigma =\sum_{i}\gamma J_{i}X_{i}\geq 0\;\text{for}i\in \left\{q,w\right\}\text{,}$$

where *γ* is the perimeter, subscripts *q* and *w* denote heat and water transfer, respectively. The entropy production is calculated for each subsystem and summed as $\sigma =\sigma_{a-m}+\sigma_{m-\text{it}}+\sigma_{\text{it}-\text{art}}+\sigma_{\text{it}-\text{ven}}$, where *σ* is the local entropy production from all subsystems, $\sigma_{a-m}$ is the local entropy production from fluxes between air-mucus subsystems, $\sigma_{m-\text{it}}$ is the local entropy production from fluxes between mucus-interstitial tissue, $\sigma_{\text{it}-\text{art}}$ is the local entropy production from fluxes between interstitial tissue-artery, and $\sigma_{\text{it}-\text{ven}}$ is the local entropy production from fluxes between interstitial tissue-vein. The local entropy production related to the air-mucus interface can be separated in two contributions from the heat flux ($J_{q}$) and from the mass flux ($J_{w}$), respectively, as $\sigma_{a-m}=\sigma_{a-m,q}+\sigma_{a-m,w}$.

The total entropy production during a breathing cycle, $\Sigma_{\text{irr}}$, is obtained by integrating *σ* over the time of the breathing cycle (inhalation and exhalation),

(3) $$\Sigma_{\text{irr}}=\int_{\text{in}}\sigma dt+\int_{\text{ex}}\sigma dt\;\geq 0,$$

where "in" stands for the time duration of inhalation and "ex" stands for the time duration of exhalation. Here, $\sigma_{a-m}$, $\sigma_{a-m,q}$, and $\sigma_{a-m,w}$ are described as,

(4) $$\int \sigma_{a-m}dt=\int \sigma_{a-m,w}dt+\int \sigma_{a-m,q}dt\text{,}$$

The explicit expressions for transport across the mucus surface are,

(5) $$\Sigma_{a-m,w}=\int \sigma_{a-m,w}dt=\int \gamma_{a}J_{w}\left(-\left(\frac{\mu_{w,m}}{T_{m}}-\frac{\mu_{w,a}}{T_{a}}\right)+h_{w,a}\left(\frac{1}{T_{m}}-\frac{1}{T_{a}}\right)\right)dt\text{,}$$

(6) $$\Sigma_{a-m,q}=\int \sigma_{a-m,q}dt=\int \gamma_{a}J_{q,a-m}^{\prime }\left(\frac{1}{T_{m}}-\frac{1}{T_{a}}\right)dt\text{,}$$

We see from Eq. 6 how temperature differences as well as differences in the chemical potential of water (which is related to relative humidity) are associated with the dissipated energy. Frictional losses due to viscous flow have been shown to be small (21). The entropy production becomes larger, the larger these differences are, and the larger the flows of heat and water. The expressions are direct measures of energy dissipation that need to be replaced in terms of food or water.

### Numerical solution procedure

The code to solve the heat and mass balance equations for the breathing cycle was originally developed to study the reindeer nose (6). In this work, the code was modified to include geometrical, histological, and physiological data for seals, as presented in Table 1. The solution procedure is verified and presented in the supporting material, section 2.

The governing equations of model II include five partial differential equations (PDEs), one for each energy balance in the five subsystems depicted in Fig. 3. Two additional PDEs were obtained for the mass balances (see Eqs. S8–S21 in the supporting material, section 1). The set of PDEs was transformed into a set of ordinary differential equations by discretization in the *z*-direction. The number of grid points in use was $N_{s}$. We decided to use $N_{s}=24$ to have a reasonable computation time, cf. Table S1, which shows the computation time for each discretization number. In each energy balance PDE, the spatial derivative, ∂T/∂z, was approximated by a fourth-order finite difference using the subroutine *dss020* (36).

The set of ordinary differential equations was solved by a MATLAB solver, *ode15s*, which solves stiff differential-algebraic equations, using backward differentiation formulas, also known as Gear’s method (37,38). We used a mass matrix that was associated with the boundary conditions listed above to obtain the temperature profile through the nose as a function of time. The simulation was running until the temperature and the total entropy production satisfied a stopping criterion. Approximately 200 cycles were needed to reach convergence (see supporting material, section 2 for specific details).

Not only iteration convergence but also grid convergence was confirmed in this study. We verified grid convergence for the subtropical seal at $T_{\text{amb}}=10$°C. The number of control volumes was determined by the discretization number $N_{s}\in \left\{6,12,24,48\right\}$. The solution of the expired air temperature was $T_{a,\text{ex}}=297.1\pm 2.83\%$ (K) while the total entropy production was $\Sigma_{\text{irr}}=0.007\pm 0.03\%$ (JK^–1^ cyc^–1^) (39) with $N_{s}=24$. Further details about grid convergence and residual analysis are given in the supporting material, section 2.

### Physical properties and physiological input

Table 1 gives the essential physiological input data and the boundary conditions used in this work. In the absence of better knowledge, the same physiological input; i.e., body mass *M*, tidal volume $V_{t}$, and breathing cycle duration $t_{\text{br}}$ is used for all seals. The data points shown by markers in Fig. 2 were used as input variables for $A_{a}$ and $\gamma_{a}$. The body temperature ($T_{\text{body}}$) was constant, but the ambient temperature ($T_{\text{amb}}$) varied. The liquid mucus layer thickness listed in Table 1 is inferred from various animals. The lining is composed of a watery solution (5–8 *μ*m) as the lower layer and the top of the lining is a viscous gel (5–10 *μ*m) (40). The total thickness of the epithelial lining fluid was estimated to be 10 *μ*m. Further physical-chemical and physiological data are described in detail in the supporting material, section 1.

### Case studies

Real and constructed seal conditions were used in this study to establish which seal nose geometry shows the highest energy efficiency. The following cases were studied.

**Figure 4:**
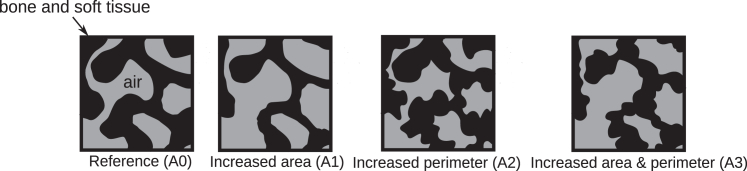
Schematic representation of the four cases with different perimeter or/and cross-sectional area for airflow (see Table 2).

1) Arctic seal (Eb) in normal life, resting in its natural habitat: in this case, the Arctic seal-specific data and boundary conditions were used, to establish characteristic properties of this seal in harsh environmental conditions (−30°C). The Arctic seal was also studied at an ambient temperature of 10°C.
2) Subtropical seal in an environmental temperature of 10°C, which would represent a cold day for a Mediterranean monk seal, Mm.
3) Subtropical seal in an Arctic environment, at −30°C: the case was constructed to investigate how a subtropical seal would in principle perform under Arctic conditions.
4) The Arctic seal at −30°C was also studied with modified $A_{a}$ and $\gamma_{a}$ (see Table 2 and Fig. 4). The perimeter ($\gamma_{a}$) and/or the cross-sectional area ($A_{a}$) were increased in steps by a factor of 1.2 for sensitivity analysis and to study this aspect of the structure-function relationship of a seal nose.

**Table 2:** Geometrical data used in the case studies

Case	A0 (Eb)	A1	A2	A3	Unit	Reference
$A_{a,\max}$	3.55 × 10^–3^	× 1.2^2^	–	× 1.2^2^	m^2^	(14)
$\gamma_{a,\max}$	11.1	–	× 1.2	× 1.2	m	(14)

Arctic seal values provide A0. The perimeter ($\gamma_{a}$) and the airspace cross-sectional area ($A_{a}$) were (artificially) increased by a factor 1.2 as indicated.

Table 2 shows the perturbations that were applied to study the effect of changes to $A_{a}$ and $\gamma_{a}$ (Fig. 4) on the nasal cavity entropy production in Arctic and subtropical seals. Changes like this could be achieved physiologically with a change in the complexity of MT structure and thickness of the nonbony layers. For example, a change in the cross-sectional area available for airflow, $A_{a}$, could be due to a change in the thickness of the mucosal layer (set to ∼200 *μ*m in the reference, A0 case, based on (27)) or of the mucus layer above that. Given that the thicknesses of these layers are estimated in our model, such perturbations allow us to assess the sensitivity of our predictions to these estimated values. The nasal mucosa of mammals can change thickness based on vascular congestion (43), so these changes in the model may also reflect physiological adjustments.

## Results and discussion

**Figure 5:**
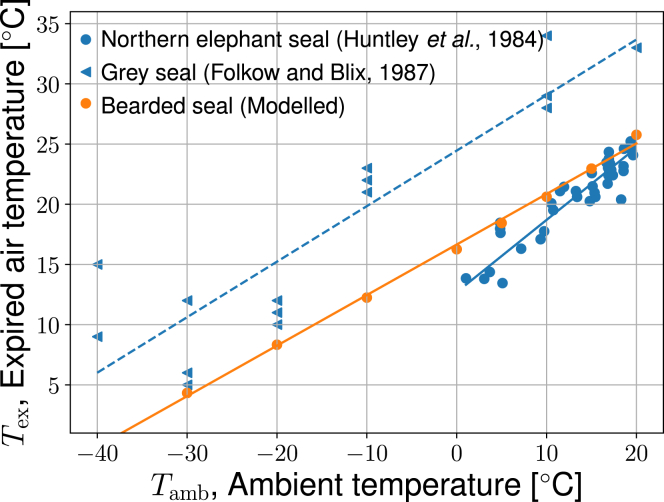
The expired air temperature of exhaled air as a function of ambient temperature, as measured in a northern elephant seal (*blue circle markers*) (22), a grey seal (*blue triangle markers*) (15), and computed by the present model using geometric data from Eb (bearded seal) (*orange markers*). Lines were fitted to experimental and modeled data by linear regression, giving $0.6T_{\text{amb}}+12.7$ (*blue solid line*), $0.46T_{\text{amb}}+24.5$ (*blue dashed line*), and $0.42T_{\text{amb}}+16.7$ (*orange line*), respectively. To see this figure in color, go online.

**Figure 6:**
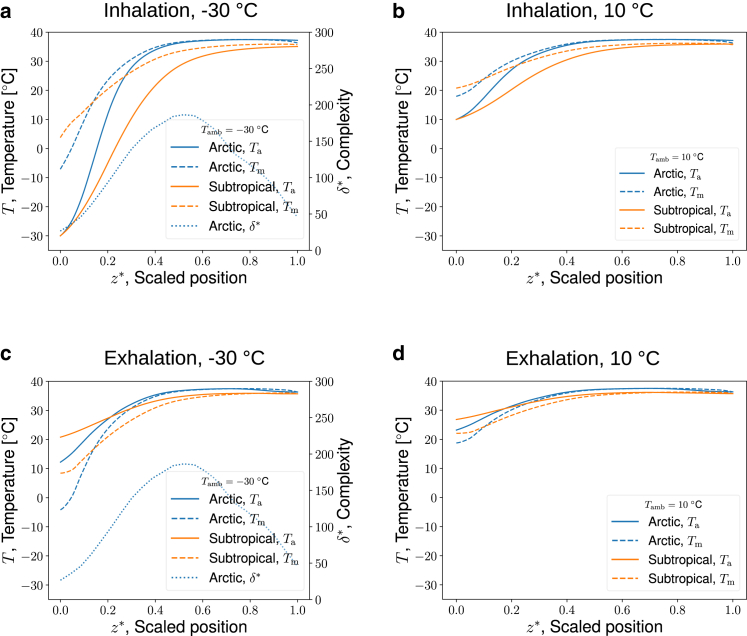
Snapshots of air temperature profiles in the MT of an Arctic seal (*blue solid line*) and a subtropical seal (*orange solid line*), (*a* and *b*) when $t^{*}=1/4$ (inhalation) and (*c* and *d*) when $t^{*}=3/4$ (exhalation). (*a* and *c*) At −30°C and (*b* and *d*) at 10°C. The dotted lines in (*a* and *c*) show the complexity factor, $\delta^{*}$, of the Arctic seal. To see this figure in color, go online.

**Figure 7:**
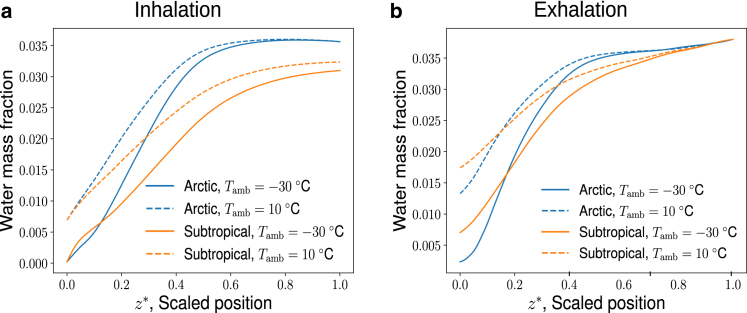
Water mass fraction profile of the air in the MT (*a*) during inhalation when $t^{*}=1/4$ and (*b*) during exhalation when $t^{*}=3/4$. Results are shown as blue lines for the Arctic seal and as orange lines for the subtropical seal. The solid lines stand for Arctic temperature conditions, −30°C, and the dashed lines stand for 10°C. To see this figure in color, go online.

**Figure 8:**
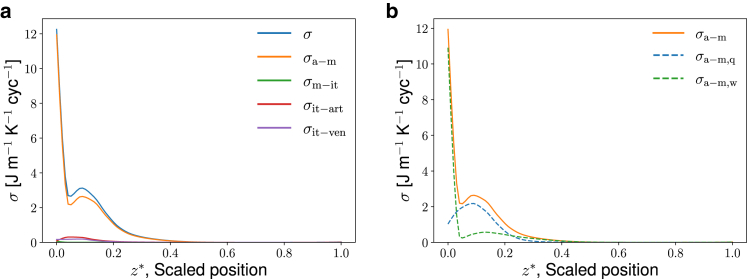
(*a*) Local entropy production for all subsystems and their sum, *σ* (*blue line*, total), $\sigma_{a-m}$ (*orange line*, air-mucus), $\sigma_{m-\text{it}}$ (*green line*, mucus-interstitial tissue), $\sigma_{\text{it}-\text{art}}$ (*red line*, interstitial tissue-artery), and $\sigma_{\text{it}-\text{ven}}$ (*purple line*, interstitial tissue-vein), of the Arctic seal at −30°C as a function of scaled position, $z^{*}$. Results apply to a single breathing cycle. (*b*) Local entropy production of air subsystem only. Each contribution and its sum are shown $\sigma_{a-m}$ (*orange line*, air-mucus), $\sigma_{a-m,q}$ (*blue dashed line*, air-mucus, heat transfer), and $\sigma_{a-m,w}$ (*green dashed line*, air-mucus, water transfer) as a function of scaled position, $z^{*}$, for the Arctic seal. To see this figure in color, go online.

**Figure 9:**
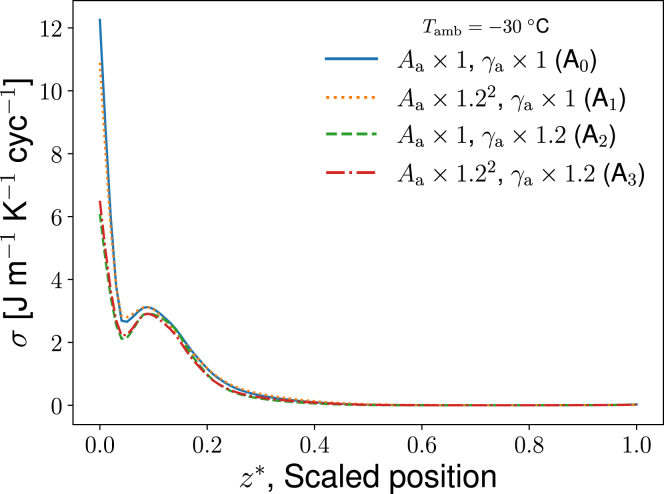
Local entropy production profile at the air-mucus interface as a function of scaled positions ($z^{*}$) in the MT of Eb, for various choices of cross-sectional area and perimeter. To see this figure in color, go online.

Results are shown in Figs. 5, 6, 7, 8, and 9 and Tables 3, 4, 5, 6, and 7, and in the video of the breathing cycle found in the supporting material, section 5. Results are shown for both species of seals under ambient temperatures −30 or 10°C.

**Table 3:** Heat and water loss in a single breathing cycle of Arctic and subtropical seals

$T_{\text{amb}}$ (°C)	Heat loss (J/cyc)		Water loss (mg/cyc)	
	Arctic	Subtropical	Arctic	Subtropical
−30	940	1364	0.04	0.14
10	492	737	0.11	0.19

**Table 4:** Heat and water recovery of the Arctic and subtropical seals

$T_{\text{amb}}$ (°C)	Heat recovery (%)		Water recovery (%)	
	Arctic	Subtropical	Arctic	Subtropical
−30	72.2	59.5	94.7	83.0
10	73.7	60.4	80.6	67.8

**Table 5:** Total entropy production ($\Sigma_{\text{irr}}$) and dissipated energy ($W_{\text{lost}}$) of the Arctic and subtropical seals for ambient temperatures ($T_{\text{amb}}$) at −30 and 10°C

$T_{\text{amb}}$ (°C)	Total entropy production ($\Sigma_{\text{irr}}$) (JK^–1^ cyc^–1^)		Energy dissipation ($W_{\text{lost}}$) (J cyc^–1^)	
	Arctic	Subtropical	Arctic	Subtropical
−30	0.025	0.041	6.0	10.0
10	0.004	0.007	1.1	2.0

**Table 6:** Total and partial entropy production (JK^–1^ cyc^–1^) of the interface between air and mucus subsystems

$T_{\text{amb}}$ (°C)	Arctic			Subtropical		
	$\Sigma_{a-m}$	$\Sigma_{a-m,q}$	$\Sigma_{a-m,w}$	$\Sigma_{a-m}$	$\Sigma_{a-m,q}$	$\Sigma_{a-m,w}$
−30	0.0220	0.0117	0.0103	0.0378	0.0201	0.0177
10	0.0034	0.0011	0.0023	0.0061	0.0019	0.0041

The contributions to the total entropy production from heat and water transport are $\Sigma_{a-m,q}$ and $\Sigma_{a-m,w}$, respectively.

**Table 7:** Sensitivity of the Arctic seal (Eb) total entropy production to changes in the complexity factor $\delta^{*}$, using hypothetical seal geometries under Arctic conditions

Total entropy production, w.r.t. A0 (%)	A1 (increased $A_{a}$)	A2 (increased $\gamma_{a}$)	A3 (increased and $\gamma_{a}$)
$\Sigma_{\text{irr}}$	−0.99	−22.9	−21.8

### Model II and experimental results

Very few experiments report nose temperature in seals: the expired air temperature was measured as a function of ambient temperature in gray seals (*Halichoerus grypus*) by Folkow and Blix (15) and in northern elephant seals (*Mirounga angustirostris*) by Huntley et al. (22). Both sets of experimental results are shown in Fig. 5 by triangles and circles, respectively. With an overlapping habitat, one might expect the results for northern elephant seal and gray seal to be more similar. However, there is some uncertainty in measurements related to single seal studies (body weight differences, etc.) and possibly also the fact that expired air temperature in seals appears to be subject to physiological (thermoregulatory) control (44). We did not attempt to use species-specific anatomical data to model the gray and elephant seals. It is nonetheless interesting to compare these experimental data with the model results we obtained for the Arctic seal, Eb.

The expired air temperature was therefore computed as a function of ambient temperature using model II with data from the Arctic seal (Eb). Boundary conditions were taken from the experiments (15). Results were not sensitive to a variation in the body temperature within 1 degree and 36°C was used. The model results, reported in Fig. 5, fall on a straight (*orange*) line with slope 0.42. The measured expired air temperature of the gray seal decreased monotonically from +20 to −30°C, with a possible minimum at the lowest temperatures. The line fitted to these results (the *dotted line*) produced a similar slope as the model, 0.46, but an offset in temperature of more than 5°C was seen (see Fig. 5). Likely reasons for the difference between the model and the gray seal results are that 1) gray seals have a less elaborate MT system than Eb (14) and 2) we do not model the air passing around the outside of the MT mass, between the MT mass and the wall, where there might be less exchange. Live seals can furthermore exercise thermoregulatory control over $T_{\text{ex}}$, which will be high under warm conditions (10–20°C) (15,44).

Our model was based on the bearded seal, Eb, rather than either the elephant or gray seals. Since Eb has a more northerly distribution than the other two species, it is perhaps surprising that our model predictions fall between the two sets of experimental data. The model predictions are sensitive to morphological and physiological variables, which likely differ between these three species; however, this issue will be discussed in more detail later.

At low temperatures, the model fails to produce the observed increase in temperature at the tip of the nose. This failure has been explained earlier as due to the vascular system around the nose tip (21). There are blood vessels for heating in this region (27), which, in combination with increased ventilation (15), probably could explain the temperature increase seen in the experimental data. Since we are not accounting for this in model II, the deviation between the model and experiments is not surprising. The experimental results for the elephant seal fall closer to model II predictions for Eb.

### Models I and II compared

Flekkøy et al. (21) made the first attempt to model nasal heat and water exchange during breathing in seals. Their model, called model I, differs from model II in several respects. In model I the blood flow is tuned to provide sufficient heat for the nose to keep from freezing, mimicking the biological control function. The air temperature was computed as the average temperature of a Poiseuille flow of air saturated with water. In model II, the air temperature is a function of the resistance to heat and mass transfer at the mucus-air interface, and a given set of boundary conditions including a set blood temperature. The relative humidity of the air is below unity at the nostrils. While model I assumes equilibrium for water (no resistance) at the air-mucus interface, model II assumes that this resistance is considerable (45). While water and heat transfer go hand in hand in model I, they can be varied independently in model II, see supporting material, section 1. For this reason, model II is more suited for an evaluation of the dissipated energy in the nose. Water and heat transport may thus in the outset contribute to the entropy production in the nose in different quantifiable ways.

### The breathing cycle

#### Temperature profiles

The temperature profiles along the nose were computed for the chosen boundary conditions, and separately for the air, mucus lining, interstitial tissue, artery, and vein subsystems. The dynamic process of breathing is shown in a video in the supporting material, section 5, Video S1. With the given transport properties and geometries, we found that the temperatures of the interstitial tissue, the arteries and veins were everywhere so close to each other that we chose to report only the temperature of the air and mucus lining. Fig. 6, *a* and *b* show snapshots of air ($T_{a}$) and mucus layer ($T_{m}$) profiles during inhalation and exhalation. Fig. 6, *a* and *c* show results for both seals under Arctic ambient conditions, while Fig. 6, *b* and *d* show both seal noses under moderate ambient temperatures.

Video S1. Video showing the variation in the temperature profiles with time during one respiration cycle

During inhalation at $t^{*}=1/4$ the air flows from left to right in Fig. 6, *a* and *b*. Since the air comes from a relatively cold and low-humidity atmosphere, the air temperature is lower than the temperature in the mucus layer. Heat is transferred from the wet mucus wall to the air in the nasal cavity due to the temperature difference, as well as due to the evaporating vapor. The temperature of the mucus layer shows a jump at the nostril end of the MT, near $z^{*}=0$ for both seals and more so at −30°C than at 10°C. During exhalation at $t^{*}=3/4$ the air flows from right to left in Fig. 6, *c* and *d*. Since the air comes from the lungs, the air temperature is higher than the temperature in the mucus layer. Heat is transferred from the air to the wet mucus wall of the nose due to the temperature difference, as well as due to the condensing vapor. The temperature of the mucus layer shows a significant change at the inlet, at $z^{*}=0.2$ for both seal conditions, and more at −30°C.

The results are sensitive to geometric variables (see Fig. 6). The mucus and the mucosa (interstitial tissue) thicknesses have an impact on the temperature profile inside the nose but not on the expired air temperature (not shown in Fig. 5). The resistance of the mucus-air interface to evaporation has a small impact on flow turbulence, and so does a variation in the heat capacity of the blood. We have therefore chosen to keep model II as presented until more experimental data become available.

By increasing the mucus thickness 50-fold, the turning point of the temperature curve will move closer to the vestibular region, while maintaining the expired air temperature at $z^{*}∼0$. Such an increase in the mucus thickness serves to increase the buffer capacity for heat and water. The boundary conditions at the inlet to the nasopharynx may also play a role. This is presently not a variable. With more accurate knowledge of the thickness of the underlying mucosa layer (interstitial tissue), which may be subject to thickening through vascular congestion, together with that of the mucus which it secretes, the precision of the computations can be increased.

The variation in the profiles is similar under both ambient temperature conditions. As we approach the nostril end ($z^{*}∼0$), the temperature difference between the air and the mucus layer increases at all conditions and for both seals. The difference is maximal closest to the nostril (see Fig. 6, *a*–*d*, *solid* and *dashed lines*), and this difference diminishes when $z^{*}$ increases toward 1. The observation means that there is sufficient time and/or amount of tissue available for adequate heat and water recovery. It is curious that the mucus temperature is lower than the air temperature during exhalation at all values of $z^{*}$ in both seals except when $z^{*}∼0.8$ for the Arctic seal (see Fig. 6, *a*–*d*). The mucus is cooled in the inhalation step, and needs to be warmed during exhalation.

The subtropical seal shows an expired air temperature higher than that of the Arctic seal at both ambient temperature conditions. We infer that the Arctic seal is better equipped to retain its heat under all conditions. We return to this issue below.

The variations in the complexity, $\delta^{*}$, across the nose, shown in Fig. 6, *a* and *c*, have a maximum at $z^{*}∼0.5$. It is interesting that the position with the maximum $\delta^{*}$ coincides with where the temperature of the air starts to change during both inhalation and exhalation. At this position, one may infer that the need for heating diminishes.

The temperature profile of the subtropical seal at 10°C changes monotonically from right to left, not far from linearly (see Fig. 6, *b* and *d*). This is unlike in the Arctic seal when $T_{\text{amb}}=-30$°C. Here, the seal nose seems to provide for a more rapid change in temperature (see Fig. 6, *a* and *c*). In particular, the temperature change is large around $z^{*}∼0.2$ in both Fig. 6, *a* and *b*.

This observation of the larger air and mucus temperature changes in the nasal cavity of the Arctic seal leads to the conclusion that the Arctic seal has a better ability to conserve body heat than the subtropical one. The ability must be ascribed to the MT morphology, as it is the only difference between the two seals within the model. These conclusions were possible because the physiological parameters were chosen to be the same and the seals were studied under the same boundary conditions.

### Water mass fraction profiles

The water mass fraction profiles in the air along the MT are presented in Fig. 7, *a* and *b* for both seal species at −30 and 10°C ambient temperature conditions. The air with 90% relative humidity comes into the nostrils ($z^{*}∼0$) and becomes saturated due to evaporation from the underlying tissues. Water mass fraction increases as the air travels toward the nasopharynx, resembling the temperature profile in Fig. 6, *a* and *b*. When we compare the water mass fraction in the two seals at the nasopharynx, at $z^{*}∼1$ we see a clear difference. The Arctic seal is able to add (and also to recover) much more water than the subtropical seal for the same ambient Arctic conditions, due to its ability to fully warm the inhaled cold air to equilibration with the body temperature. The blue lines are always above the orange lines at ($z^{*}∼1$). This is despite the fact that the absolute humidity of the environment differs (it depends exponentially on the ambient temperature). The amount of water vapor adsorbed by the mucosa depends greatly on the geometry of the seal’s MT. During exhalation in Fig. 7 *b* we see a bigger change in water mass fraction for Arctic seals than for subtropical seals when $z^{*}=1\to 0$. This is because relatively more water can be absorbed into the mucosal walls in the nasal cavity of the Arctic seal than in the subtropical seal, which also reflects a different water adsorption ability of the two species.

### Heat and water recovery

The heat and water loss and recovery were computed for a single breathing cycle from the profiles of Figs. 6 and 7 presented previously, using the equations in the supporting material, section 1.

During inhalation, water and heat are transferred to the inspired air in the nose from the mucus layer. During exhalation, water and heat are transferred in the opposite direction. The losses computed during one breathing cycle are shown in Table 3 for two ambient temperatures, while Table 4 gives the recovery in percentage of the amount that would have been lost if no recovery took place.

Under both temperature conditions, we see that the Arctic seal has a smaller loss of heat and water. Recovery of heat and water is better for the Arctic than the subtropical seal under both conditions. In particular, at −30°C, the Arctic seal shows a very high water recovery, 94.7% (see Table 4). Most of the water added to the air during inhalation is then recovered during exhalation. The fact that the saturated water vapor pressure increases exponentially with temperature helps explain why the relative recovery of water is higher at −30°C than at 10°C (see Tables 3 and 4).

The heat leaving the nose per cycle of a subtropical seal is, according to the data of Table 3, ∼45% larger than for the Arctic seal when both are under −30°C ambient temperature conditions. For these reasons, the MT structure of the Arctic seal is superior to that of the subtropical seal for both temperature conditions investigated in terms of both heat and water conserved.

Since the main difference, when both seals are exposed to the same ambient temperature, is their MT structure, these results point to the importance of this in the adaptation of seals to the Arctic. We did not investigate water and heat loss at higher environmental temperatures, but it should be recognized that, on a warm day, a seal may benefit from respiratory heat loss, although it does possess other (primarily behavioral) avenues to enhance heat dissipation (e.g., entering water).

### Entropy production and energy dissipation

The entropy production and the energy dissipated by heat and mass transfer were computed for one breathing cycle. The total entropy production and its contributions are presented in Tables 5 and 6, respectively. We see that there is a variation in the contributions to the total and local entropy production between the species and by changing ambient conditions. This variation can be linked directly to the MT structure and to the complexity of the MT structure. The more complex the MT is, the lower is the total entropy production of the breathing cycle. Recalling that entropy production is a direct measure of the dissipated energy which must be replenished with energy from food, we may conclude that seals with a more complex MT will enjoy an advantage in cold conditions.

Consider first the total entropy production and the corresponding dissipated energy in the seal nose, as shown for both species’ MT structures in Table 5. The total entropy production applies to the whole nose undergoing one breathing cycle. It is obtained by integrating the local entropy production over time and space. We see from Table 5 that the energy dissipation in subtropical seals is much greater than in Arctic seals at −30°C (10.0 vs. 6.0 J cyc^−1^). The Arctic seal is, relatively speaking, wasting much less energy than subtropical seals do when they both are put under Arctic ambient conditions. The corresponding numbers (2.0 vs. 1.1) are much smaller and more comparable in size when the ambient temperature reaches 10°C. These results are directly traceable to the more elaborate MT structure of the Arctic seal. The value of the total entropy production can be broken down into contributions to give more insight into the differences observed in Table 5. We have distinguished between five contributions; transfer between air and mucus lining, $\sigma_{a-m}$, mucosa and interstitial tissue, $\sigma_{m-\text{it}}$, interstitial tissue and arteries $\sigma_{\text{it}-\text{art}}$ or veins $\sigma_{\text{it}-\text{ven}}$ (see methods and materials for details). Their values are reported for the Arctic seal under Arctic conditions, −30°C, in Fig. 8 *a*. Other ambient conditions give similar trends.

Among all contributions in Fig. 8 *a* to the total value in Table 5, the local entropy production of the air-mucus interface, $\sigma_{a-m}$, is clearly the largest. It is followed by contributions from the mucus-interstitial tissue interface, $\sigma_{m-\text{it}}$, the interstitium-artery contact, $\sigma_{\text{it}-\text{art}}$, and the interstitium-vein contact, $\sigma_{\text{it}-\text{ven}}$. The $\sigma_{\text{it}-\text{art}}$ and $\sigma_{\text{it}-\text{ven}}$ did not contribute significantly to the total entropy production, *σ*, and are from now on neglected. In other words, the contributions that affect the total and local entropy production most are the water and heat exchange processes between air and mucosa. The relative importance of heat and water recovery can also be studied by separating $\sigma_{a-m}$ into contributions; i.e., from heat transport ($\sigma_{a-m,q}$) and water transport ($\sigma_{a-m,w}$). We found that the high *local* entropy production at the nostril end of the nasal cavity is due mostly to water transport. The contribution is largest right at the entrance (see Fig. 8, *a* and *b*), and can be explained by the chosen difference in chemical potential of water at the inlet (the deviation of the water vapor pressure from its saturation value). The contribution from heat transport dominates the entropy production when $z^{*}>0.05$. The total entropy production depends on the ambient temperature (see Table 6). At −30°C, heat transport explains a large part of the total entropy production, but the contribution from water exchange is of the same order of magnitude. At temperatures near 10°C, water transport is relatively more important. Neither of the two contributions appears more important than the other.

The dissipation is sensitive to changes in the MT structure. To see this we applied model II to three variations in the nasal cavity structure of the Arctic seal (Eb). The cross-sectional area available to airflow ($A_{a}$ in case A0) was first increased by a factor of $1\text{.}2^{2}$ (case A1), the perimeter $\gamma_{a}$ was next increased by a factor of 1.2 (case A2), and finally area and perimeter were both increased (case A3). These cases are directly related to a change in the complexity of the MT structure, as $\delta^{*}=\gamma_{a}/\sqrt{A_{a}}$ shows. Some of these changes could occur physiologically by the seal inflating or deflating its nasal mucosa through changes in blood vessel congestion. The effects of these changes on the entropy production are shown in Table 7 and Fig. 9.

Table 7 shows the contribution to the total entropy production from the various transport processes through the nose in the four cases described in Table 2. We see that the entropy production is sensitive to both airway area and perimeter but to a varying degree. A change in the perimeter has by far the larger influence on entropy production.

Fig. 9 shows the entropy production from heat and water transport between the air and mucus subsystems for each of the four cases in Table 2. The entropy production in the air and mucus of the Arctic seal (*blue solid line*), reference case A0, changes negligibly when we change the cross-sectional airway area only (*yellow dotted line*). An increase in the perimeter leads to a reduction of 22.9% in the entropy production (*green dashed line*). By again increasing the area (*red dash-dotted line*), there is a marginally smaller decrease in entropy production. This suggests that the perimeter of the MT structure is an important property for a seal living in the Arctic regarding energy dissipation. Relatively less energy is needed to compensate for energy dissipation in a nose with a large MT perimeter.

To obtain an overall perspective on the model results, we computed the energy dissipation on a macro level and compared it with the results for other animals from several references. Note that we need to consider the length of a nose from each animal species to calculate this energy dissipation by integrating the local entropy production plotted in Figs. 8 *a* and 9. A subtropical seal at 10°C ambient condition loses, according to model II, 2.0 J per breathing cycle, for a cycle of 3.1 s (see Table 1). This translates to 2.3 kJ/h or 55.9 kJ/day. For the reindeer nose (6), the corresponding number was 1.4 kJ/h or 34 kJ/day. Seals are known to have irregular breathing cycles featuring periodic apnea, so a simple averaging of a continuous cycle over the day may not be appropriate. Resting seals typically maintain breathing rates of ∼10 breaths per min (15,44), which would give energy dissipation values in the order of ∼1 kJ/h or ∼23 kJ/day.

More accurate anatomical data concerning mucus and interstitial tissue thicknesses would be helpful in improving the predictions of model II. The Arctic seal Eb enjoys good heat and water recovery based on its elaborate MT structure. This is likely also to be true for other polar seals, which have similarly complex MT. Our data allow for a quantitative analysis of the hypothesis proposed by Mason et al. (14). The results are sensitive to geometric variables, of which we have direct information only about the bony MT structures. With more accurate knowledge of the morphology of the overlying soft tissue and mucus layer, the tidal volume, and pharynx boundary conditions, the precision in the computations can be increased. For now, we see that a 50-fold increase in the thickness of the mucus shifts the turning point of the temperature profile toward the nose opening.

## Conclusion

We have presented the time-dependent temperature and water mass fraction profiles of the air inspired and expired by the Arctic and subtropical seals, Eb and Mm, living in the Arctic and moderate temperature conditions, respectively. The breathing process was simulated for a quasi-1D representation of the MT structure. Anatomical and physiological parameters were used with various ambient boundary conditions to solve the mass, energy, and entropy balances. The set of equations, called model II, was able to predict, with reasonable accuracy, the temperature of the expired air measured as a function of ambient temperature. We found that the MT structure gives the Arctic seal (Eb) an advantage over the subtropical seal (Mm) in the recovery of both heat and water under Arctic ambient conditions. The more complex MT structure helps the Arctic seal to maintain a temperature similar to body temperature within the nasopharynx. An in-depth analysis of the energy dissipation of the breathing cycle showed that the perimeter of the MTs could play an essential role in limiting energy dissipation, especially at low ambient temperatures. An increase in perimeter leads to a relatively large decrease in total energy dissipation (entropy production).

## Data and code availability

The url for nose calculation code is: https://github.com/hyejeonc/nose-calculations.

## Author contributions

H.L.C. adapted the computer program to deal with seal noses, validated the program, and carried out all calculations. M.J.M. provided new anatomical data based on CT scans. S.K. and N.K. designed the research plan and devised the methods. H.L.C., S.K., and N.K. wrote the first manuscript draft. L.P.F. and M.J.M. provided insight into biological structure and function. All authors discussed the meaning of the results in a biological context and helped edit the last versions of the manuscript.

## Supporting material

Document S1. Figures S1–S5 and Tables S1–S4

Document S2. Article plus supporting material
