# Supplementary material for: Structure-function relationships in the nasal cavity of Arctic and subtropical seals
Source: Biophys J. 2023 Dec 14;122(24):4686–98. doi: 10.1016/j.bpj.2023.11.012 (PMC10754691; doi:10.1016/j.bpj.2023.11.012)
Supplement: Document S1. Figures S1–S5 and Tables S1–S4 [file mmc1.pdf]

Biophysical Journal, Volume *122*

Supplemental information

Structure-function relationships in the nasal cavity of Arctic and sub-tropical seals

Hyejeong L. Cheon, Signe Kjelstrup, Nataliya Kizilova, Eirik G. Flekkøy, Matthew J. Mason, and Lars P. Folkow

Supplemental information

November 3, 2023

This document contains equations used in the model, numerical characteristics of solutions and more detailed information on heat and water fluxes.

1 Model equations

1.1 Balance equations

The energy balance of a cylindrical control volume (CV) is described as [1],

$$\begin{bmatrix}\text{Rate of energy}\\ \text{accumulation}\\ \text{in CV}\end{bmatrix} = \begin{bmatrix}\text{Net inflow of}\\ \text{energy by heat flux}\\ \text{in z-direction}\end{bmatrix} + \begin{bmatrix}\text{Net inflow of}\\ \text{energy by heat flux}\\ \text{in r-direction}\end{bmatrix} + \begin{bmatrix}\text{Change of energy}\\ \text{due to changed amount of}\\ \text{components within CV}\end{bmatrix} \tag{S1}$$

Here, we rewrite all heat fluxes (J_q) into measurable heat fluxes (J'_q) as [2, p.26, 29, 225],

$$J_\mathrm{q,r} = {J'}_\mathrm{q,r} + \sum_i (h_i - h_\mathrm{ref}) J_{i,r}, \tag{S2}$$

$$J_\mathrm{q,z} = {J'}_\mathrm{q,z} + \sum_i (h_i - h_\mathrm{ref}) J_{i,z}, \tag{S3}$$

where J_q is the total heat flux, J_i, is the flux of ith component, h_i is the enthalpy of i component and h_ref is the reference enthalpy, and subscripts r and z specify the direction: therefore, the second term on the RHS (right-hand side) in Eqs. S2 and S3 above shows the enthalpy flux. We reformulate the term on the LHS (left-hand side) of Eq. S1 for the air by the cross-sectional area, A_a, the perimeter, γ_a, and the width of the CV, Δz, as,

$$\begin{bmatrix}\text{Rate of energy}\\ \text{accumulation}\\ \text{in CV}\end{bmatrix} = (A_\mathrm{a}\Delta z)\rho_\mathrm{a} c_\mathrm{p,a}\frac{dT_\mathrm{a}}{dt}, \tag{S4}$$

where ρ_a is the density of the air, T_a is the temperature of the air, t is the time and $c_\mathrm{p,a}$ is the specific heat capacity of the air subsystem. Subscripts m, it, art and ven stand for the mucus, interstitial tissue, artery and vein subsystems, respectively, from now on. The first term on the RHS of Eq. S1 is,

$$\begin{aligned}\begin{bmatrix}\text{Net inflow of}\\ \text{energy by heat flux}\\ \text{in z-direction}\end{bmatrix} &= -A_\mathrm{a} J_\mathrm{q,z}\\ &= -A_\mathrm{a}({J'}_\mathrm{q,z} + \sum_i (h_i - h_\mathrm{ref}) J_{i,z})\\ &= -A_\mathrm{a}\left(\frac{F_\mathrm{a}}{A_\mathrm{a}} C_\mathrm{p,a}\Delta_\mathrm{z} T_\mathrm{a} + \frac{F_\mathrm{a}}{A_\mathrm{a}}\sum_i h_i \Delta_\mathrm{z} w_i\right),\end{aligned} \tag{S5}$$

where F_a is the total mass flow in z-direction, which h_i is the specific enthalpy of the ith component and $\Delta_\mathrm{z} w_i$ is the mass fraction of the component i change in z-direction which is the ratio of the ith component mass to the total mass of the air including the ith component. The second term on the RHS in Eq. S1 is,

$$\begin{bmatrix}\text{Net inflow of}\\ \text{energy by heat flux}\\ \text{in r-direction}\end{bmatrix} = -(\gamma_\mathrm{a}\Delta \mathrm{z})J_{\mathrm{q,r}}$$

$$= -\gamma_\mathrm{a}\Delta \mathrm{z}(J'_{\mathrm{q,r}} + \sum_i (h_i - h_\mathrm{ref})J_{\mathrm{i,r}})$$

$$= -\gamma_\mathrm{a}\Delta \mathrm{z}(J'_{\text{q,a-m}} + J_\mathrm{w}\sum_i (h_i - h_\mathrm{ref})w_i)$$

$$= -\gamma_\mathrm{a}\Delta \mathrm{z}(J'_{\text{q,a-m}} + J_\mathrm{w}(h_\mathrm{w,a} - h_\mathrm{a})), \tag{S6}$$

where $J'_{\text{q,a-m}}$ is the measurable heat flux from the air to the mucus subsystem and J_w is the water mass flux from the air to the mucus lining which appears as positive for condensation and negative for evaporation. Note that there is only one mass flux for a component, water vapor, which is $J_1 = J_\mathrm{w}$ and $w_1 = 1.0$. The amount of components in the air has changed over time and this is shown in the third term of RHS in Eq. S1. This is rewritten as,

$$\begin{bmatrix}\text{Change of energy}\\ \text{due to changed amount of}\\ \text{components within CV}\end{bmatrix} = \rho_\mathrm{a}(A_\mathrm{a}\Delta z)\sum_i h_i \frac{dw_i}{dt}. \tag{S7}$$

By Eqs. S4-S7, energy balance of the air subsystem is,

$$(A_\mathrm{a}\Delta z)\rho_\mathrm{a} c_\mathrm{p,a}\frac{dT_\mathrm{a}}{dt} = -\left(F_\mathrm{a} c_\mathrm{p,a}\Delta_\mathrm{z} T_\mathrm{a} + F_\mathrm{a}\sum_\mathrm{i} h_i \Delta_\mathrm{z} w_i\right)$$

$$- \gamma_\mathrm{a}\Delta z(J'_{\text{q,a-m}} + J_\mathrm{w}(h_\mathrm{w,a} - h_\mathrm{a}))$$

$$+ \rho_\mathrm{a} A_\mathrm{a}\Delta z \sum_i h_i \frac{dw_i}{dt}. \tag{S8}$$

We change Δz to ∂z and dt to ∂t here with an assumption that Δz is infinitesimal. We omit subscript r and z for a convenience, then,

$$A_\mathrm{a}\rho_\mathrm{a} c_\mathrm{p,a}\frac{\partial T_\mathrm{a}}{\partial t} = -\left(F_\mathrm{a} c_\mathrm{p}\frac{\partial T_\mathrm{a}}{\partial t} + F_\mathrm{a}\sum_\mathrm{i} h_i \frac{\partial w_i}{\partial z}\right) - \gamma_\mathrm{a}(J'_{\text{q,a-m}} + J_\mathrm{w}(h_\mathrm{w,a} - h_\mathrm{a})) + \rho_\mathrm{a} A_\mathrm{a}\sum_i h_i \frac{\partial w_i}{\partial t}$$

$$= -F_\mathrm{a} c_\mathrm{p,a}\frac{\partial T_\mathrm{a}}{\partial t} - \gamma_\mathrm{a} J'_{\text{q,a-m}} - \gamma_\mathrm{a} J_\mathrm{w}(h_\mathrm{w,a} - h_\mathrm{a}) + A_\mathrm{a}\rho_\mathrm{a}\sum_i h_i \frac{\partial w_i}{\partial t} - F_\mathrm{a}\sum_i h_i \frac{\partial w_i}{\partial z}. \tag{S9}$$

The energy balance of the mucus subsystem is,

$$\begin{bmatrix}\text{Rate of energy}\\ \text{accumulation}\\ \text{in CV}\end{bmatrix} = \begin{bmatrix}\text{Net inflow of}\\ \text{energy by heat flux}\\ \text{in r-direction,}\end{bmatrix} + \begin{bmatrix}\text{Net inflow of}\\ \text{energy by source}\end{bmatrix}. \tag{S10}$$

Here, the heat flux from the air to mucus, the heat flux from mucus to the interstitial tissue and water supplied from the underlying tissues are considered. This is rewritten as,

$$A_\mathrm{m}\rho_\mathrm{m} c_\mathrm{p,m}\frac{\partial T_\mathrm{m}}{\partial t} = \gamma_\mathrm{m}(J'_{\text{q,a-m}} + \sum_i (h_i - h_\mathrm{ref})J_i) - \gamma_\mathrm{m} J'_{\text{q,m-it}} + \gamma_\mathrm{m} J_\mathrm{m}(h_\mathrm{w,m} - h_\mathrm{ref})$$

$$= \gamma_\mathrm{m}(J'_{\text{q,a-m}} + J_\mathrm{w}(h_\mathrm{w,a} - h_\mathrm{ref})) - \gamma_\mathrm{m} J'_{\text{q,m-it}} + \gamma_\mathrm{m} J_\mathrm{m}(h_\mathrm{w,m} - h_\mathrm{ref})$$

$$= \gamma_\mathrm{m}(J'_{\text{q,a-m}} + J_\mathrm{w}(h_\mathrm{w,a} - h_\mathrm{w,m})) - \gamma_\mathrm{m} J'_{\text{q,m-it}}, \tag{S11}$$

$$
\begin{aligned}
A_{\mathrm{m}}\rho_{\mathrm{m}}c_{\mathrm{p,m}}\frac{\partial T_{\mathrm{m}}}{\partial t} &= \gamma_{\mathrm{m}}(J'_{\text{q,a-m}} + \sum_i (h_i - h_{\mathrm{ref}})J_i) - \gamma_{\mathrm{m}}J'_{\text{q,m-it}} + \gamma_{\mathrm{m}}J_{\mathrm{m}}(h_{\mathrm{w,m}} - h_{\mathrm{ref}}) \\
&= \gamma_{\mathrm{m}}(J'_{\text{q,a-m}} + J_{\mathrm{w}}(h_{\mathrm{w,a}} - h_{\mathrm{ref}})) - \gamma_{\mathrm{m}}J'_{\text{q,m-it}} + \gamma_{\mathrm{m}}J_{\mathrm{m}}(h_{\mathrm{w,m}} - h_{\mathrm{ref}}), \\
\rho_{\mathrm{m}}c_{\mathrm{p,m}}\frac{\partial T_{\mathrm{m}}}{\partial t} &= \frac{1}{A_{\mathrm{m}}}(\gamma_{\mathrm{m}}(J'_{\text{q,a-m}} + J_{\mathrm{w}}(h_{\mathrm{w,a}} - h_{\mathrm{w,m}})) - \gamma_{\mathrm{m}}J'_{\text{q,m-it}}) \\
&= \frac{\gamma_{\mathrm{m}}}{A_{\mathrm{m}}}((J'_{\text{q,a-m}} + J_{\mathrm{w}}(h_{\mathrm{w,a}} - h_{\mathrm{w,m}})) - J'_{\text{q,m-it}}) \\
&= \frac{1}{d_{\mathrm{m}}}((J'_{\text{q,a-m}} + J_{\mathrm{w}}(h_{\mathrm{w,a}} - h_{\mathrm{w,m}})) - J'_{\text{q,m-it}}),
\end{aligned} \tag{S12}
$$

where ρ_{m} is the density of mucus and $c_{\mathrm{p,m}}$ is the specific heat capacity of the mucus subsystem. Other mucus subsystem variables (subscript m) are equivalent to those in the air subsystem, defined above. Note that mucus water flux $J_{\mathrm{m}} = -J_{\mathrm{w}}$ from mass balance of the mucus subsystem where J_{w} is the mass flux of water generated from underlying tissues. The energy balance of the interstitial tissue is described as,

$$
A_{\mathrm{it}}\rho_{\mathrm{it}}c_{\mathrm{p,b}}\frac{\partial T_{\mathrm{it}}}{\partial t} = \gamma_{\mathrm{m}}J'_{\text{q,m-it}} - \gamma_{\mathrm{art}}J'_{\text{q,it-art}} - \gamma_{\mathrm{ven}}J'_{\text{q,it-ven}}, \tag{S13}
$$

where T_{it} is the temperature of the interstitial tissue, $J'_{\text{q,it-art}}$ is the measurable heat flux from the interstitial tissue to the artery in the r-direction, $J'_{\text{q,it-ven}}$ is the measurable heat flux from the interstitial tissue to the vein in the r-direction, ρ_{it} is the density of the blood and $c_{\mathrm{p,b}}$ is the specific heat capacity of blood. Energy balance of artery and vein subsystems is described as,

$$
A_{\mathrm{art}}\rho_{\mathrm{b}}c_{\mathrm{p,b}}\frac{\partial T_{\mathrm{art}}}{\partial t} = -F_{\mathrm{art}}c_{\mathrm{p,b}}\frac{\partial T_{\mathrm{art}}}{\partial z} + \gamma_{\mathrm{art}}J'_{\text{q,it-art}}, \tag{S14}
$$

where A_{art} is the cross-sectional area of artery, F_{art} is the blood flow in the arterial blood, T_{art} is the temperature of the arterial blood, γ_{art} is the perimeter of the artery subsystem. For the vein subsystem,

$$
A_{\mathrm{ven}}\rho_{\mathrm{b}}c_{\mathrm{p,b}}\frac{\partial T_{\mathrm{ven}}}{\partial t} = -F_{\mathrm{ven}}c_{\mathrm{p,b}}\frac{\partial T_{\mathrm{ven}}}{\partial z} + \gamma_{\mathrm{ven}}J'_{\text{q,it-ven}}, \tag{S15}
$$

where A_{ven} is the cross-sectional area of the vein, F_{ven} is the blood flow in the vein, T_{ven} is the temperature of the venous blood and γ_{ven} is the perimeter of the vein subsystem. The mentioned cross-sectional areas and perimeters of interstitial tissue, mucus lining, artery and vein subsystems are described specifically in the following section.

Similarly, mass balance of a cylindrical CV is described as follows. Mass balance of the air without water vapor (dry air) and water vapor are written as,

$$
\frac{\partial(A_a\rho_{\mathrm{dry}})}{\partial t} = -\frac{\partial F_{\mathrm{dry}}}{\partial z}, \tag{S16}
$$

$$
\frac{\partial(A_{\mathrm{a}}\rho_{\mathrm{w,a}})}{\partial t} = -\frac{\partial F_{\mathrm{w,a}}}{\partial z} + J_{\mathrm{w}}\gamma_{\mathrm{a}}, \tag{S17}
$$

where ρ_{dry} is the density of the dry air, $\rho_{\mathrm{w,a}}$ is the density of the water vapour, F_{dry} is the mass flow of the dry air and $F_{\mathrm{w,a}}$ is the mass flow of the water vapor. ρ_{a} is a sum of ρ_{dry} and $\rho_{w,a}$ ($\rho_{\mathrm{a}} = \rho_{\mathrm{dry}} + \rho_{w,a}$), and F_{a} is a sum of F_{dry} and $F_{\mathrm{w,a}}$ ($F_{\mathrm{a}} = F_{\mathrm{dry}} + F_{\mathrm{w,a}}$). Mass balance of the water liquid in the mucus layer is described as,

$$
\frac{\partial(A_{\mathrm{m}}\rho_{\mathrm{m}})}{\partial t} = J_{\mathrm{w}}\gamma_{\mathrm{a}} + J_{\mathrm{m}}\gamma_{\mathrm{a}} = 0. \tag{S18}
$$

Mass balance for the interstitial tissue, artery and vein all are 0 on the RHS due to the assumption that $\rho_{\mathrm{b}} = \rho_{\mathrm{it}} = \textit{constant}$ and $F_{\mathrm{art}} = -F_{\mathrm{ven}} = \textit{constant}$. This is written as,

$$
\frac{\partial(A_{\mathrm{it}}\rho_{\mathrm{it}})}{\partial t} = 0, \tag{S19}
$$

$$
\frac{\partial(A_{\mathrm{art}}\rho_{\mathrm{b}})}{\partial t} = -\frac{\partial F_{\mathrm{art}}}{\partial z} = 0, \tag{S20}
$$

$$\frac{\partial(A_{\text{ven}}\rho_{\text{b}})}{\partial t} = -\frac{\partial F_{\text{ven}}}{\partial z} = 0. \tag{S21}$$

1.2 Equations of transport

Non-equilibrium thermodynamics [3] defines the fluxes (J) and conjugate forces (X) from the entropy production as,

$$\sigma = \sum_i J_i X_i \geq 0 \qquad \text{for} \quad i \in \{q, w\}. \tag{S22}$$

Eq. 2 in the main text is the local entropy production regarding quasi-1D interpretation [4, 5], therefore, this is obtained by multiplying the perimeter (γ); $\sigma = \sum_i \gamma J_i X_i$. Subscripts q and w denote heat and water transfer, respectively. By the linear force-flux relations,

$$X_k = \sum_j R_{kj} J_j \qquad \text{for} \quad k, j \in \{q, w\}, \tag{S23}$$

where specific values for the resistivities, R_{kj}, are listed in Section 4. The entropy production is calculated for each subsystem and summed as $\sigma = \sigma_{\text{a-m}} + \sigma_{\text{m-it}} + \sigma_{\text{it-art}} + \sigma_{\text{it-ven}}$ where σ is the local entropy production from all subsystems, $\sigma_{\text{a-m}}$ is the local entropy production from fluxes between air-mucus subsystems, $\sigma_{\text{m-it}}$ is the local entropy production from fluxes between mucus-interstitial tissue, $\sigma_{\text{it-art}}$ is the local entropy production from fluxes between interstitial tissue-artery and $\sigma_{\text{it-ven}}$ is the local entropy production from fluxes between interstitial tissue-vein. The local entropy production related to the air-mucus interface can be separated in two contributions, from the heat flux and from the mass flux, respectively, as $\sigma_{\text{a-m}} = \sigma_{\text{a-m,q}} + \sigma_{\text{a-m,w}}$.

In most subsystems, there is only heat flux between two subsystems, i.e., mucus-interstitial tissue, interstitial tissue-artery and interstitial tissue-vein. We write this as:

$$\frac{1}{T_j} - \frac{1}{T_i} = R_{\text{q},i-j} J_{\text{q},i-j}, \tag{S24}$$

where T_j and T_i are the temperatures of subsystem j and i, respectively, and $R_{\text{q},i-j}$ is the overall thermal transport coefficient. $R_{\text{q},i-j}$ is the sum of resistances in series [6, p.104],

$$R_{\text{q},i-j} = R^{\text{conv}}_{\text{q},i} + R^{\text{interf}}_{\text{q},i-j}, \tag{S25}$$

$$R^{\text{conv}}_{\text{q},i-j} = \frac{1}{T_i^2 h_i}, \tag{S26}$$

where $R^{\text{conv}}_{\text{q},i}$ is the convective thermal transport coefficient of subsystem i, $R^{\text{interf}}_{\text{q},i-j}$ is the conductive thermal transport coefficient at the interface between two subsystems, i and j, and h_i is the convective heat transfer coefficient of system i. Note that $R^{\text{conv}}_{\text{q},i}$ is non-zero for subsystems of the artery and vein but not for the interstitial tissue and the mucus, and we will deal with the thermal transfer coefficient related convective flow for the air later. The h_i is determined by Nusselt number, Nu, which is a ratio between heat transfer by convection and heat transfer by conduction:

$$h_i = \frac{\text{Nu}\lambda}{D_i}. \tag{S27}$$

The Nu is 3.66 for convective and laminar blood flow in a cylinder [7, p.676], λ is the thermal conductivity of blood and D_i is the hydraulic diameter of a channel of subsystem i [8]:

$$D_i = \frac{4\gamma_i}{A_i}. \tag{S28}$$

Here γ_i is the perimeter of subsystem i and A_i is the cross-sectional area of subsystem i.

For the subsystem related to the coupled transport of heat and mass, for example, the air and mucus, the coupled force-flux relation is described as regarding non-equilibrium thermodynamics [2, p.100],

$$\frac{1}{T_m} - \frac{1}{T_a} = R_{\mathrm{qq,a\text{-}m}} J'_{\mathrm{q,a\text{-}m}} + R_{\mathrm{q}\mu,\mathrm{a\text{-}m}} J_{\mathrm{w}}, \tag{S29}$$

$$-\left(\frac{\mu_{\mathrm{w,m}}}{T_{\mathrm{m}}} - \frac{\mu_{\mathrm{w,a}}}{T_{\mathrm{a}}}\right) + h_{\mathrm{w,a}}\left(\frac{1}{T_{\mathrm{m}}} - \frac{1}{T_{\mathrm{a}}}\right) = R_{\mu\mathrm{q,a\text{-}m}} J'_{\mathrm{q,a\text{-}m}} + R_{\mu\mu,\mathrm{a\text{-}m}} J_{\mathrm{w}}, \tag{S30}$$

where $\mu_{\mathrm{w,m}}$ is the chemical potential of the mucus, the same as for the liquid water, $\mu_{\mathrm{w,a}}$ is the chemical potential of the water vapor in the air and a subscript q stands for the heat transport and μ is for the mass transport. Onsager reciprocal relation gives the fact that the coupling coefficients are equal, therefore, the number of independent coefficients are reduced to 3 which are $R_{\mathrm{qq,a\text{-}m}}$, $R_{\mathrm{q}\mu,\mathrm{a\text{-}m}} = R_{\mu\mathrm{q,a\text{-}m}}$ and $R_{\mu\mu,\mathrm{a\text{-}m}}$. The same approach introduced previously for the heat transport is applied here also,

$$R_{\mathrm{qq,a\text{-}m}} = R^{\mathrm{conv}}_{\mathrm{qq,a}} + R^{\mathrm{interf}}_{\mathrm{qq,a\text{-}m}} + R^{\mathrm{conv}}_{\mathrm{qq,m}}, \tag{S31}$$

$$R^{\mathrm{conv}}_{\mathrm{qq,a\text{-}m}} = \frac{1}{T_{\mathrm{a}}^2 h}, \tag{S32}$$

and $R^{\mathrm{interf}}_{\mathrm{qq,a\text{-}m}}$, $R^{\mathrm{interf}}_{\mu\mu,\mathrm{a\text{-}m}}$ and $R^{\mathrm{interf}}_{\mu\mathrm{q,a\text{-}m}}$ are tabulated in Appendix C which is the finding from [9]. To obtain h, heat transfer coefficient (or film coefficient), Nu is calculated by the Chilton-Colburn analogy [7, p.441]. We assume that the airflow in the maxilloturbinate is in the turbulent regime. This is because the turbinate structure of the seal is much more complex than that of the humans, as discussed [10, 11]. The Nu is,

$$\mathrm{Nu} = 0.125 f \mathrm{Re}\mathrm{Pr}^{1/3}, \tag{S33}$$

where f is the friction factor, Re is the Reynolds number and Pr is the Prandtl number. The f is estimated from a study of human nasal cavity [12] as,

$$f = \frac{47.78}{\mathrm{Re}}(1 + 0.127\mathrm{Re}^{0.489}), \tag{S34}$$

and Reynolds number (Re) depends on the airflow and hydraulic diameter: the value obtained during simulation was ∼1000. This is a variable varying in time with the breathing dynamics. Flekkøy et al. found a slightly smaller and constant Re for flow between parallel plates [13]. We assume here that convection can be ignored, which means that,

$$R_{\mu\mathrm{q,a\text{-}m}} = R^{\mathrm{interf}}_{\mu\mathrm{q,a\text{-}m}}. \tag{S35}$$

The overall mass transfer coefficient is described as,

$$R_{\mu\mu,\mathrm{a\text{-}m}} = R^{\mathrm{conv}}_{\mu\mu,\mathrm{a}} + R^{\mathrm{interf}}_{\mu\mu,\mathrm{a\text{-}m}} + R^{\mathrm{conv}}_{\mu\mu,\mathrm{m}}, \tag{S36}$$

and

$$R^{\mathrm{conv}}_{\mu\mu,\mathrm{a\text{-}m}} = \frac{1}{T_{\mathrm{a}} k_{\mathrm{c}}} \frac{\partial \mu_{\mathrm{w,a}}}{\partial \rho_{\mathrm{w,a}}}, \tag{S37}$$

where k_{c} is the convective mass transfer coefficient which is calculated from the Sherwood number for turbulent air flow [7, p.757, 761] as,

$$k_{\mathrm{c}} = \frac{\mathrm{Sh} D_{\mathrm{w,a}}}{D_{\mathrm{a}}}, \tag{S38}$$

$$\mathrm{Sh} = 0.023\mathrm{Re}^{0.8}\mathrm{Sc}^{1/3}, \tag{S39}$$

where Sc is the Schmidt number. Sc is defined as,

$$\mathrm{Sc} = \frac{\nu}{D_{\mathrm{AB}}}, \tag{S40}$$

where ν is the kinematic viscosity which is the dynamic viscosity divided by the density of the air, and D_{AB} is the mass diffusivity of the air [7, p.724, 761].

We also tested the Nusselt and Sherwood numbers in Eqs. S33 and S39 in the laminar flow regime, because we do not know whether the airflow within the seal maxilloturbinates is turbulent or laminar [10, 11]. We found that the temperature profile in Fig. 6 of the main text does not vary significantly if the expressions for laminar flow as follows are used [7, p.780]:

$$\mathrm{Nu} = 1.86 \left(\frac{\mathrm{RePr} D_{\mathrm{a}}}{L} \right)^{1/3} \left(\frac{\mu_{\mathrm{a}}}{\mu_{\mathrm{wall}}} \right), \tag{S41}$$

$$\mathrm{Sh} = 3.66, \tag{S42}$$

where μ_{wall} is the dynamic viscosity of maxilloturbinate wall and μ_{a} is the dynamic viscosity of the air.

1.3 Tissue characteristics

We briefly describe here how we calculate the composition of tissue for seals. We describe the input variables related to the cross-sectional area of the air channels and the perimeter of the mucus lining. Due to a lack of experimental data, all values are approximated by corresponding values estimated for a reindeer [10, 14, 15, 16].

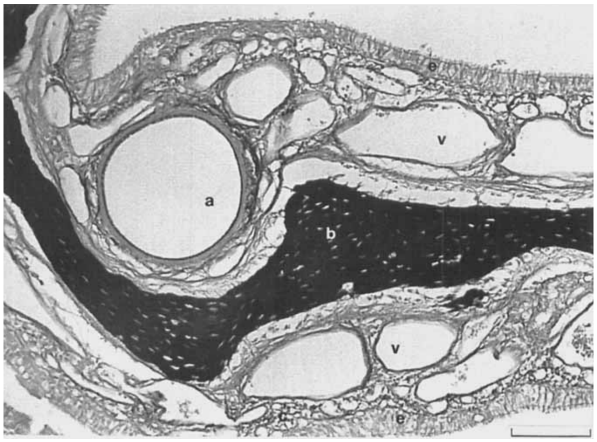

Figure S1: Cross-section of a turbinate branch section from a grey seal, *Halichoerus grypus*. The scale bar is 100 μm. a stands for the lumen of an artery, v labels the lumina of veins, b refers to the bony part of the maxilloturbinate, e refers to the epithelium and the white area above the epithelial layer is the air cavity. The image is reproduced with permission of Folkow et al. [17], © 1988 Wiley.

Position (reduced scale by L)	0.2	0.4	0.6	0.8	1.0
$\hat{\gamma}_{\mathrm{art}}$ [m/m]	0.77	0.62	0.32	0.33	0.44
$\hat{A}_{\mathrm{art}}$ [m^2/m]	5.8 $\times 10^{-5}$	3.9 $\times 10^{-5}$	1.2 $\times 10^{-5}$	1.5 $\times 10^{-5}$	3.8 $\times 10^{-5}$
r_{art}	0.053	0.035	0.019	0.015	0.048
$\hat{\gamma}_{\mathrm{ven}}$ [m/m]	3.31	2.76	1.70	1.51	2.20
$\hat{A}_{\mathrm{ven}}$ [m^2/m]	6.7 $\times 10^{-4}$	6.1 $\times 10^{-4}$	1.9 $\times 10^{-4}$	2.3 $\times 10^{-4}$	5.5 $\times 10^{-4}$
r_{ven}	0.42	0.38	0.25	0.20	0.26

Table S1: Cross-sectional area and perimeter of arteries and veins at various positions along the reindeer nose [14], where the nose length is L. Fraction (r_{art} and r_{ven}) here refers to the fraction of the area which is occupied by arteries from the total cross-sectional area of the tissue. Cross-sectional area ($\hat{A}$) and perimeter ($\hat{\gamma}$) are proportional to the perimeter of the air cavity, γ_{a}. Units in brackets are therefore described as per meter. All variables related to the interstitial tissue are computed from Eqs. S45-S47.

The thickness of the mucus lining of the seal was set to 10 μm according to [15, 16]. These authors assumed that the thickness of the mucus lining is similar in all animal species. The perimeter of the mucus lining is similar to the perimeter of the air cavity. The cross-sectional area of the mucus lining then is equal to the product of the perimeter and the thickness,

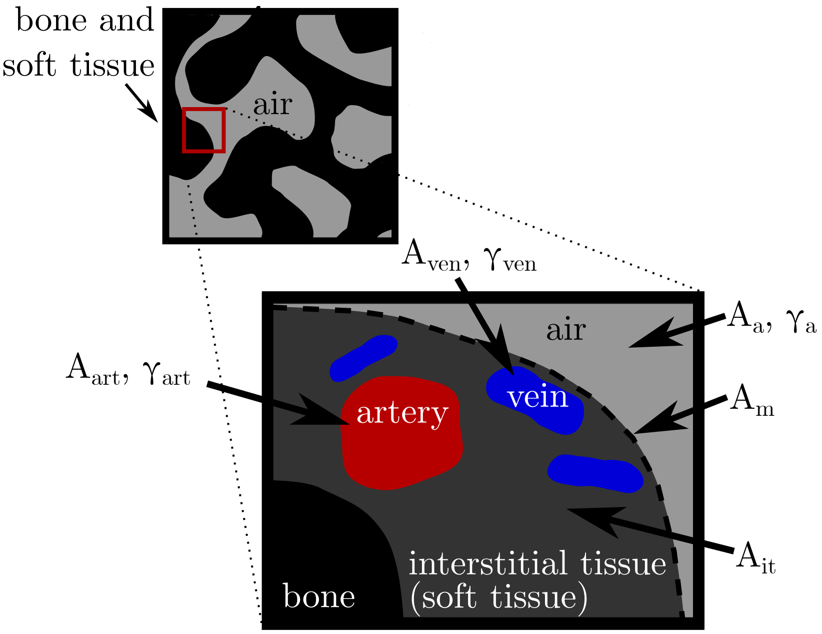

Figure S2: Cross-section of maxilloturbinates with variables listed in Table S1.

$$A_{\mathrm{m}} = \gamma_{\mathrm{m}} d_{\mathrm{m}}, \tag{S43}$$
$$\approx \gamma_{\mathrm{a}} d_{\mathrm{m}}. \tag{S44}$$

Here γ_{m} is the perimeter of mucus lining, d_{m} is the thickness of the mucus lining, 10 μm, and γ_{a} is the perimeter of the air cavity. Arteries and veins and denoted with subscripts a and v in Fig. S1 and illustrated in Fig. S2 also. The measured cross-sectional areas and perimeters are shown in Table S1. We calculated the approximated areas and perimeters of artery and vein from,

$$\gamma_{\mathrm{art}} = \gamma_{\mathrm{a}} \hat{\gamma}_{\mathrm{art}}, \tag{S45}$$
$$A_{\mathrm{art}} = \gamma_{\mathrm{a}} \hat{A}_{\mathrm{art}}, \tag{S46}$$

where γ_{art} is the perimeter of artery, and A_{art} is the cross-sectional area of artery. γ_{a} is the perimeter of the air cavity, $\hat{\gamma}_{\mathrm{art}}$ is the perimeter of artery per unit length and $\hat{A}_{\mathrm{art}}$ is the cross-sectional area of artery per unit length. The cross-sectional area and perimeter of the vein were obtained in the same manner.

The cross-section of the interstitial tissue contains arteries and veins, but the area of the avascular interstitial tissue can be obtained by subtracting the cross-sectional area of the arteries and veins from the total cross-sectional area of the tissue. The cross-sectional area of the interstitial tissue is known if r_{art} and r_{ven} are known, see Table S1. Variables in Table S1 are related as,

$$A_{\mathrm{it}} = (A_{\mathrm{it}} + A_{\mathrm{art}} + A_{\mathrm{ven}}) r_{\mathrm{it}},$$
$$A_{\mathrm{it}} = (A_{\mathrm{it}} + A_{\mathrm{a}} \hat{A}_{\mathrm{art}} + A_{\mathrm{a}} \hat{A}_{\mathrm{ven}})(1 - (r_{\mathrm{art}} + r_{\mathrm{ven}})),$$
$$A_{\mathrm{it}}(r_{\mathrm{art}} + r_{\mathrm{ven}}) = (A_{\mathrm{a}} \hat{A}_{\mathrm{art}} + A_{\mathrm{a}} \hat{A}_{\mathrm{ven}})(1 - (r_{\mathrm{art}} + r_{\mathrm{ven}})),$$
$$A_{\mathrm{it}} = \frac{(A_{\mathrm{a}} \hat{A}_{\mathrm{art}} + A_{\mathrm{a}} \hat{A}_{\mathrm{ven}})(1 - (r_{\mathrm{art}} + r_{\mathrm{ven}}))}{r_{\mathrm{art}} + r_{\mathrm{ven}}}, \tag{S47}$$

where r_{it} is the ratio of the cross-sectional area of the interstitial tissue to the cross-sectional area of the whole tissue, while r_{art} is the cross-sectional area ratio of the artery to the tissue, and r_{ven} is the cross-sectional area ratio of the vein to the tissue. This is summarized below:

- $\frac{\text{Cross-sectional area of artery}}{\text{Perimeter of air cavity}} = \hat{A}_{\mathrm{art}}$. Therefore we can estimate cross-sectional area of artery, A_{art}.

- $\frac{\text{Perimeter of artery}}{\text{Perimeter of air cavity}} = \hat{\gamma}_{\text{art}}$. Therefore we can estimate perimeter of artery, γ_{art}.
- $\frac{\text{Cross-sectional area of vein}}{\text{Perimeter of air cavity}} = \hat{A}_{\text{ven}}$. Therefore we can estimate cross-sectional area of vein, A_{ven}.
- $\frac{\text{Perimeter of vein}}{\text{Perimeter of air cavity}} = \hat{\gamma}_{\text{ven}}$. Therefore we can estimate perimeter of vein, γ_{ven}.

1.4 Heat and water recovery

The absolute amount of net water vapor we need to add to the expired air after completing a breathing cycle is denoted $M_{\text{w,add}}$. The value is calculated as the amount of water vapor entering the nose from the lungs during exhalation minus the amount of water vapor entering the nose ($M_{\text{w,add}}$) from the ambient air during inhalation:

$$M_{\text{w,add}} = \int_{\text{ex}} (-F_{w,a})_{\text{z=L}} dt - \int_{\text{in}} (F_{w,a})_{\text{z=0}} dt, \tag{S48}$$

where $F_{w,a}$ is the mass flow of water vapor in nasal air. The absolute amount of water recovered ($M_{\text{w,rec}}$) in the nose is the amount of water vapor entering the nose from the lungs during exhalation minus the amount of air flowing out through the nostrils during exhalation. This is obtained from,

$$M_{\text{w,rec}} = \int_{\text{ex}} (-F_{w,a})_{\text{z=L}} dt - \int_{\text{ex}} (-F_{w,a})_{\text{z=0}} dt. \tag{S49}$$

The fractional recovery of water is thus $M_{\text{w,rec}}/M_{\text{w,add}}$.

The heat recovery can be obtained similarly. The heat added to the nasal cavity (Q_{add}) is the sum of the heat that is necessary to warm up the inhaled air to the body temperature and the heat that is necessary to evaporate the water added during inhalation. This is:

$$Q_{\text{add}} = \int_{\text{in}} F_{\text{a}} c_{\text{p,a}} (T_{\text{body}} - T_{\text{amb}}) dt + M_{\text{w,add}} h_{\text{w,lat}}, \tag{S50}$$

where F_{a} is air mass flow and $c_{\text{p,a}}$ is specific heat capacity of air as 1003 $\text{Jkg}^{-1}\text{K}^{-1}$ and $h_{\text{w,lat}}$ is the latent heat of evaporation of water as 2264 kJkg^{-1}. The amount of recovered heat during exhalation (Q_{rec}) is given by the sum of the sensible heat that is subtracted from the air during exhalation, and the latent heat that is released by condensing of water,

$$Q_{\text{rec}} = \int_{\text{ex}} F_{\text{a}} c_{\text{p,a}} (T_{\text{body}} - T_{\text{ex}}) dt + M_{\text{w,rec}} h_{\text{w,lat}}, \tag{S51}$$

where T_{ex} is the expired air temperature and the fractional recovery of heat is calculated by $Q_{\text{rec}}/Q_{\text{add}}$. For further details on the recovery of water and heat, we refer to the literature [10, 18].

1.5 Input data related to thermodynamic properties

All variables related to physiological and thermodynamic properties are listed in Table S2. The input variables are used in most of the equations of the main part of the work.

Table S2: Variables relating to physiological and thermodynamic properties.

Symbol	Description	Value	Unit	Reference
$h_{\mathrm{w,lat}}$	Latent heat of evaporation	2264	$\mathrm{kJkg^{-1}}$	[10, 19]
$c_{\mathrm{p,a}}$	Specific heat capacity of air	1003	$\mathrm{J\ kg^{-1}\ K^{-1}}$	[7, 10]
$c_{\mathrm{p,b}}$	Specific heat capacity of blood	3620	$\mathrm{J\ kg^{-1}\ K^{-1}}$	[20]
ρ_{b}	Density of blood	1000	$\mathrm{kg\ m^{-3}}$	[10]
k_{b}	Thermal conductivity of blood	0.50	$\mathrm{J\ m^{-1}\ s^{-1}\ K^{-1}}$	[10]
$c_{\mathrm{p,m}}$	Specific heat capacity of mucus	4186	$\mathrm{J\ kg^{-1}\ K^{-1}}$	[10]
ρ_{m}	Density of mucus	1000	$\mathrm{kg\ m^{-3}}$	[10]
k_{m}	Thermal conductivity of mucus	0.60	$\mathrm{J\ m^{-1}\ s^{-1}\ K^{-1}}$	[10]
d_{m}	Mucus thickness	1.0×10^{-5}	m	[21, 22]
F_{b}	Mass flow rate of blood	2.2×10^{-4}	$\mathrm{kg\ min^{-1}}$	[10]

2 Numerical verification of the solution

2.1 Grid size and residual analysis

The effect of grid size and its effect on variables' residuals were investigated. The length of the nose was divided into a N_s number of control volumes. The air temperature profile, T_a, and the entropy production profile were computed with varying N_s. The temperature profile and the local entropy production are presented in Fig. S3a and b as a function of N_s. We see that results have not converged for $N_\mathrm{s} = 6$. Figs. S3a and b both imply that N_s should be larger than 12 to obtain grid convergence. We therefore chose 24 for the proper grid discretization number.

Specifically, the error regarding grid convergence index (GCI) is calculated based on [23, 24, 25]. GCI is an index to determine mesh refinement. By calculation processes introduced in [23, 24], we obtained an error band of the grid convergence of the solution as 2.83 % for the expired air temperature and 0.03 % for total entropy production. This is described as,

$$T_\mathrm{a,ex} \approx 297.1 \pm 2.83\ \%, \tag{S52}$$

$$\Sigma_\mathrm{irr} \approx 0.007 \pm 0.03\ \%. \tag{S53}$$

Residuals of the total entropy production (Σ_irr), the air temperature for a cycle (T_a), the expired air temperature ($T_\mathrm{a,ex}$) and the water content for a cycle (w_a) were computed for various iteration numbers. The residual of a value of any parameter A at the ith cycle is given by,

$$\text{Residual of } A = \frac{|A^{i+1} - A^{i}|}{|A^{2} - A^{1}|}, \tag{S54}$$

for matrices, i.e., w_a and T_a, we replace the absolute value by matrix norm, $||...||_2$. In our results, a residual in the range $10^{-1.7} \sim 10^{-3.8}$ was considered to be sufficient (see Fig. S4). The residuals of Σ_irr, T_a, $T_\mathrm{a,ex}$ and w_a started to oscillate after about 180 cycles for temperatures at 283 K. From this, we infer that a number of iterations larger than 200 is needed.

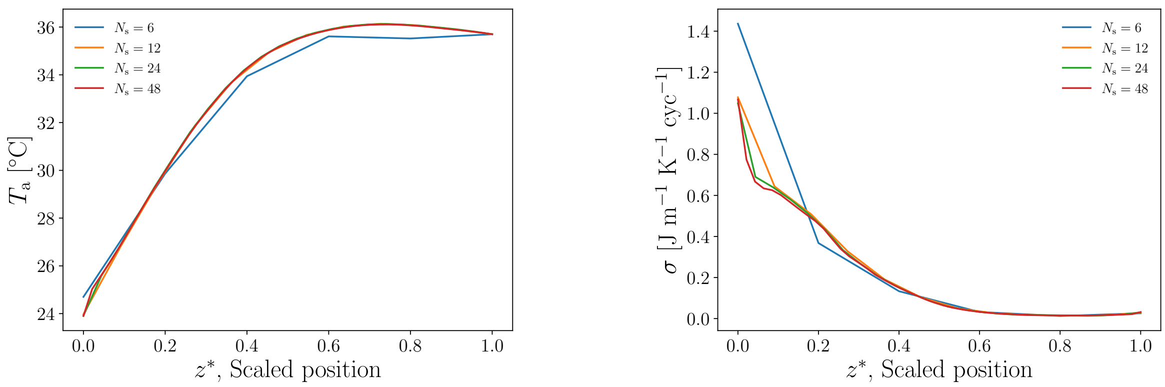

(a) (b)

Figure S3: Time-averaged (a) air temperature profile and (b) local entropy production of the subtropical seal at 10 °C ambient temperature as a function of scaled position, for discretization numbers $N_s \in \{6, 12, 24, 48\}$. Time-averaging was done during the last half of the breathing cycle, referred to as exhalation.

The model is time-dependent and the discretization in time (time step) is decided by the MATLAB Integrated routine 'ode15s'. In the 'ode15s' routine, the optimal time step is decided within a range of user-defined minima in every cycle (a single respiration cycle).

2.2 Computation time

The total time required to complete one simulation set with a discretization number in space, $N_s \in \{6, 12, 24, 48\}$, is listed in Table S3. The value $N_\mathrm{s} = 24$ gives results very near grid convergence, still with a reasonable computation time.

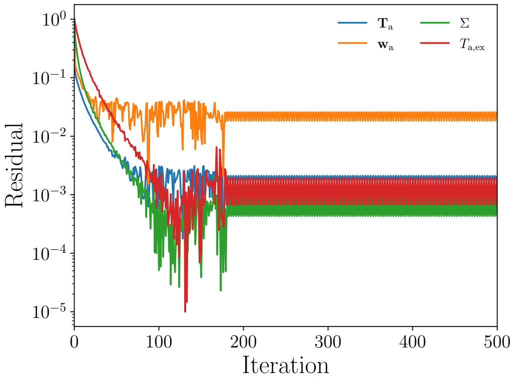

Figure S4: Residual of total entropy production (Σ), air temperature (T_a), expired air temperature during exhalation ($T_{a,ex}$) and water mass fraction (w_a) in a cycle as a function of iteration number at $T_{amb} = 10$ °C.

Table S3: Discretization step and corresponding computation time with 10 °C and the geometry of the subtropical seal's turbinate.

Discretization in space	Computation time (dd:hh:mm)
$N_s = 6$	10:03:54
$N_s = 12$	10:08:26
$N_s = 24$	11:09:36
$N_s = 48$	12:21:55

3 Heat and water fluxes

Through the video of temperature profiles described in Fig.6-7 of the main text and Section 5, we have shown the temperature profiles of each subsystem. In this section, we present the heat and water transfer between subsystems in terms of flux profiles at -30 °C.

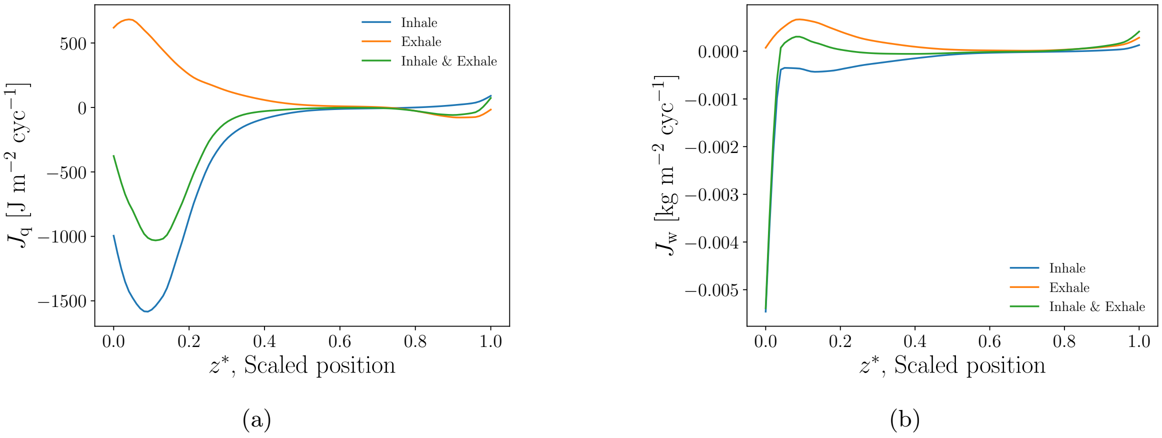

Figure S5: (a) Heat flux as a function of scaled position, z^*, averaged over inhalation, exhalation and both. (b) Water flux as a function of position, z^*, averaged over inhalation, exhalation and both. Inhalation (blue line) captures flux profiles during inhalation only, and exhalation (orange line) captures flux profiles during exhalation only. Inhale & Exhale (green line) shows time-averaged profiles over both inhalation and exhalation. Both fluxes are from the Arctic seal at -30 °C.

When cold air enters the inlet of the nose, heat is transferred from the mucus layer to the air, which will warm up the cold air and protect the lungs. This heat transfer is shown as a negative heat flux (blue line) in Fig. S5a. During exhalation, the absolute value of the flux shows a maximum around $z^* \sim 0.03$, which means that heat transfer takes place in a region behind the inlet of the nasal maxilloturbinate, and not only exactly at the inlet. The heat flux during exhalation is positive for $z^* < 0.7$ (see orange line); in contrast, heat flux during inhalation appears negative. The averaged heat flux during a total breathing cycle shows a maximum at $z^* \sim 0.1$.

In Fig. S5b, mass flux is negative during inhalation, representing water transfer from the mucus lining to air (see blue line). During exhalation, however, the mass flux is positive in the given z^* domain and water in the air is transferred back to the mucus lining (see orange line). In other words, water vapor in the saturated air from the lungs is liquefied in the nose. The averaged flux during a whole respiration cycle is large at the nostril but close to zero at $z^* > 0.2$.

These results show directly how heat and mass transfer occur between subsystems when ambient air enters and exits the nose. The fluxes determine the local entropy production, which accordingly is large near the nostril, and decreases steeply at $z^* \to 0.03$ in Fig. 8a of the main text. Variations in the local entropy production profile along the nose position have their origin in the interplay of heat and water transfer.

We examined the sensitivity of the fluxes and the local entropy production to changes in resistivity coefficients and mucus thickness. With a larger mucus thickness, the heat and mass fluxes increased significantly at the maximum. The entropy production also broadened in the nose's anterior part and became less concentrated at the nostril (not shown).

4 Transfer coefficients

Interface transfer coefficients (known as interface resistivities) were used to describe heat and water transfer. Their values are listed in Table S4. We used coefficients taken from Wilhelmsen et al. [9].

Temperature [K]	R_{qq} [m^{2}s/JK]	$R_{\mu q}$ [m^{2}s/kgK]	$R_{\mu\mu}$ [Jm^{2}s/kg^{2}K]
260	4.24E-07	3.68E-02	4.73E+03
265	3.56E-07	3.16E-02	4.17E+03
270	2.98E-07	2.71E-02	3.66E+03
275	2.49E-07	2.32E-02	3.22E+03
280	2.08E-07	1.98E-02	2.82E+03
285	1.73E-07	1.68E-02	2.46E+03
290	1.43E-07	1.43E-02	2.14E+03
295	1.18E-07	1.20E-02	1.86E+03
300	9.66E-08	1.01E-02	1.60E+03
305	7.86E-08	8.42E-03	1.37E+03
310	6.35E-08	6.97E-03	1.17E+03
315	5.10E-08	5.74E-03	9.94E+02
320	4.07E-08	4.69E-03	8.37E+02

Table S4: Interface transfer coefficients related to water-evaporation.

5 Temperature profiles of a breathing cycle (video)

This section describes the details of a video showing the temperature profile at any moment of time in the breathing cycle, based on the model derived in this study.

The video shows the temperature profiles of the five subsystems of the Arctic seal, Eb, under Arctic conditions (-30 °C) at various moments. The upper panel of the video shows the temperature profiles which are indicated by the red vertical line on the panel below. The panel below shows the air flow rate variation during the breathing cycle. The left half describes the inhalation, and the other half describes exhalation. The flow rate of air is at its maximum value at $t^* = 1/4$ and $3/4$.

We consider the temperature profiles at various times in a breathing cycle. At the start of the inhalation, the -30 °C air enters the nostril. As a result, the air temperature near the nostril end

($z^* < 0.05$) is lower than the water temperature in the mucus lining. The temperature profiles of mucus, interstitial tissue, artery and vein subsystems are almost in thermal equilibrium. The air temperature shows a deviation due to the given boundary condition, $z^* \sim 0$. For further discussion, see the main text.

At the end of the inhalation ($t^* \to 0.5$), the temperature of the air, mucus lining, interstitial tissue and vein are also almost at equilibrium. The change in the temperature profile of the air is larger than the variation of water temperature in the mucus lining. This can be expected considering that the thermal capacity of water is much larger than that of the air. When the inhalation is over, and the exhalation begins, the temperature of the air is higher than the temperature of the mucus lining ($t^* > 0.5$). The warm air comes out of the lungs, and moves toward the nostril; heat and water vapor are adsorbed by the mucus.

References

[1] Jakobsen, H. A., 2008. Chemical reactor modeling. *Multiphase Reactive Flows* .

[2] Kjelstrup, S., D. Bedeaux, E. Johannessen, and J. Gross, 2017. Non-equilibrium thermodynamics for engineers. World Scientific.

[3] Kjelstrup, S., and D. Bedeaux, 2008. Non-equilibrium thermodynamics of heterogeneous systems. World Scientific.

[4] Johannessen, E., and S. Kjelstrup, 2004. Minimum entropy production rate in plug flow reactors: An optimal control problem solved for SO2 oxidation. *Energy* 29:2403–2423.

[5] Magnanelli, E., S. B. B. Solberg, and S. Kjelstrup, 2019. Nature-inspired geometrical design of a chemical reactor. *Chemical Engineering Research and Design* 152:20–29.

[6] Incropera, F. P., D. P. DeWitt, T. L. Bergman, A. S. Lavine, et al., 1996. Fundamentals of heat and mass transfer, volume 6. Wiley New York.

[7] Cengel, Y. A., H. Pérez, et al., 2004. Heat transfer: a practical approach. Tata McGraw Hill Co.

[8] Mason, M. J., L. M. Wenger, Ø. Hammer, and A. S. Blix, 2020. Structure and function of respiratory turbinates in phocid seals. *Polar Biology* 43:157–173.

[9] Wilhelmsen, Ø., T. T. Trinh, A. Lervik, V. K. Badam, S. Kjelstrup, and D. Bedeaux, 2016. Coherent description of transport across the water interface: From nanodroplets to climate models. *Physical Review E* 93:032801.

[10] Magnanelli, E., Ø. Wilhelmsen, M. Acquarone, L. P. Folkow, and S. Kjelstrup, 2017. The nasal geometry of the reindeer gives energy-efficient respiration. *Journal of Non-Equilibrium Thermodynamics* 42:59–78.

[11] Solberg, S. B. B., S. Kjelstrup, E. Magnanelli, N. Kizilova, I. L. C. Barroso, M. Acquarone, and L. P. Folkow, 2020. Energy efficiency of respiration in mature and newborn reindeer. *Journal of Comparative Physiology B* 190:509–520.

[12] Zamankhan, P., G. Ahmadi, Z. Wang, P. K. Hopke, Y.-S. Cheng, W. C. Su, and D. Leonard, 2006. Airflow and deposition of nano-particles in a human nasal cavity. *Aerosol science and technology* 40:463–476.

[13] Flekkøy, E. G., L. P. Folkow, S. Kjelstrup, M. J. Mason, and Ø. Wilhelmsen, 2023. Thermal modeling of the respiratory turbinates in arctic and subtropical seals. *Journal of Thermal Biology* 103402.

[14] Casado Barroso, I. L., 2014. The ontogeny of nasal heat exchange structures in Arctic artiodactyles. Master's thesis, UiT Norges arktiske universitet.

[15] Frederick, C., J. Morris, J. Kimbell, K. Morgan, and P. Scherer, 1994. Comparison of four biologically based dosimetry models for the deposition of rapidly metabolized vapors in the rodent nasal cavity. *Inhalation Toxicology* 6:135–135.

[16] Morgan, K. T., X.-Z. Jiang, D. L. Patterson, and E. A. Gross, 1984. The nasal mucociliary apparatus: correlation of structure and function in the rat. *American Review of Respiratory Disease* 130:275–281.

[17] Folkow, L., A. Blix, and T. Eide, 1988. Anatomical and functional aspects of the nasal mucosal and ophthalmic retia of phocid seals. *Journal of Zoology* 216:417–436.

[18] Blix, A. S., and H. K. Johnsen, 1983. Aspects of nasal heat exchange in resting reindeer. *The Journal of physiology* 340:445–454.

[19] Oliver, J. E., 2008. Encyclopedia of world climatology. Springer Science & Business Media. https://doi.org/10.1007/1-4020-3266-8_124.

[20] Rodríguez de Rivera, P. J., M. Rodríguez de Rivera, F. Socorro, G. M. Callicó, J. A. Calbet, and M. Rodríguez de Rivera, 2021. Heat flow, heat capacity and thermal resistance of localized surfaces of the human body using a new calorimetric sensor. *Journal of Thermal Analysis and Calorimetry* 1–14.

[21] Bush, M. L., C. B. Frederick, J. S. Kimbell, and J. S. Ultman, 1998. A CFD–PBPK hybrid model for simulating gas and vapor uptake in the rat nose. *Toxicology and applied pharmacology* 150:133–145.

[22] Kaulbach, H. C., M. V. White, Y. Igarashi, B. K. Hahn, and M. A. Kaliner, 1993. Estimation of nasal epithelial lining fluid using urea as a marker. *Journal of allergy and clinical immunology* 92:457–465.

[23] Computational Fluid Dynamics (CFD) Verification and Validation Web Site of the NPARC Alliance. https://www.grc.nasa.gov/www/wind/valid/tutorial/spatconv.html.

[24] Castedo, R., C. Reifarth, A. P. Santos, J. Losada, L. M. López, M. Chiquito, and J. M. Mancilla, 2019. Application of grid convergence index to shock wave validated with LS-DYNA and ProsAir. *Ingeniería e Investigación* 39:20–26.

[25] Roache, P. J., 1998. Verification and validation in computational science and engineering, volume 895. Hermosa Albuquerque, NM.
